# Recent Research Progress on the Chemical Constituents, Pharmacology, and Pharmacokinetics of *Alpinae oxyphyllae* Fructus

**DOI:** 10.3390/molecules29163905

**Published:** 2024-08-18

**Authors:** Junfa Liao, Xueying Zhao

**Affiliations:** School of Basic Medical Sciences, Heilongjiang University of Chinese Medicine, 24 Heping Road, Harbin 150040, China; junfaliao2024@126.com

**Keywords:** *Alpinae oxyphyllae* fructus, phytochemistry, chemical constituents, pharmacology, pharmacokinetics

## Abstract

*Alpinae oxyphyllae* fructus (AOF), the dried mature fruit of *Alpinia oxyphylla* Miquel of the Zingiberaceae family, shows many special pharmacological effects. In recent years, there has been an abundance of research results on AOF. In this paper, the new compounds isolated from AOF since 2018 are reviewed, including terpenes, flavonoids, diarylheptanoids, phenolic acid, sterols, alkanes, fats, etc. The isolation methods that were applied include the microwave-assisted method, response surface method, chiral high-performance liquid chromatography–multiple reaction monitoring–mass spectrometry (HPLC-MRM-MS) analytical method, ultra-high-performance liquid chromatography–quadrupole–electrostatic field orbitrap high-resolution mass spectrometry (UPLC-Orbitrap-HRMS) method, ultra-high-performance liquid chromatography–tandem mass spectrometry (UPLC-MS/MS) method, hot water leaching method, ethanol leaching method, and so on. Additionally, the pharmacological effects of AOF found from 2018 to 2024 are also summarized, including neuroprotection, regulation of metabolic disorders, antioxidant activity, antiapoptosis, antiinflammatory activity, antidiabetic activity, antihyperuricemia, antiaging, antidiuresis, immune regulation, anti-tumor activity, renal protection, hepatoprotection, and anti-asthma. This paper provides a reference for further research on AOF.

## 1. Introduction

*Alpinae oxyphyllae* fructus (AOF) is the dried mature fruit of *Alpinia oxyphylla* Miquel of the Zingiberaceae family. *Alpinia oxyphyllae* (AO) is usually produced in Hainan, Guangdong, Guangxi, Yunnan, and other tropical and subtropical regions of China, and Hainan has become the authentic production area of *Alpinia oxyphylla* because of its favorable ecological environment and climatic conditions [1]. AOF is usually used for its medicinal parts. According to the *Chinese Pharmacopoeia*, AOF has a specific aroma, has a pungent and slightly bitter taste, and belongs to the spleen and kidney meridian [2]. In the past few decades, researchers have conducted a large number of studies on AOF, including on its chemical composition and pharmacological effects. Zhang et al. summarized the chemical composition and pharmacological effects of AOF before 2018 in detail, showing that AOF has many pharmacological effects, including neuroprotective, antidiabetic, antidiarrheal, antioxidant, anti-inflammatory, antiallergic, antitumor, antidiuretic, etc., which are applied in the treatment of neurological, urinary, and gastrointestinal system diseases [3]. In recent years, with the development of separation methods and research techniques, many researchers have isolated many new compounds from AOF, and new progress has been made in the studies of its pharmacological effects. With a view to further provide a reference for the research of AOF in the field of traditional Chinese medicine (TCM), we systematically summarize the research progress of the newly isolated chemical constituents, isolation methods, pharmacological effects, pharmacokinetics, and other aspects of AOF by collating the latest relevant literature since 2018.

## 2. Active Components of AOF

In recent years, many new technologies have been widely used to isolate and characterize the active components of AOF, and a wealth of research results have been achieved, which has promoted research on the chemical composition of AOF. Many scholars have used the microwave-assisted method, the response surface method, and ultrasonic extraction to extract compounds such as flavonoids, terpenoids, and polysaccharides from AOF, and many have applied HPLC-MRM-MS analysis and UPLC-Q-Orbitrap HRMS to characterize and quantify these compounds [4,5,6,7,8].

In the research progress on AO, most studies focused on AOF. Ying et al. analyzed the bioactive constituents of the leaves, roots, and fruits of *Alpinia oxyphylla* Miquel by using UPLC-MS/MS [9]. Duan et al. used hot water extraction, ethanol extraction, and ultrasound-assisted extraction to effectively extract polyphenols from the hulls of AOF, and the antioxidative activity of polyphenols extracted by ultrasonic-assisted extraction was the strongest [10].

In this paper, we summarize the new compounds that have been isolated from AOF in the past 5 years; a total of 85 new compounds have been obtained, which include terpenes, flavonoids, diarylheptanoids, and others.

### 2.1. Terpenes

**Figure 1:**
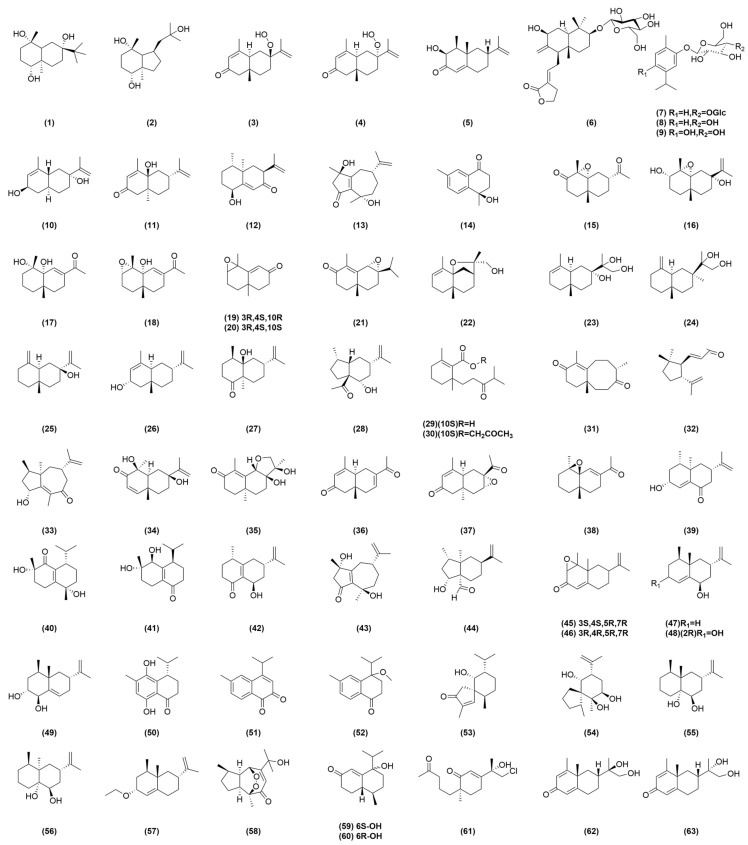
Structures of terpene compounds isolated from AOF.

Terpenoids, also known as isoprenoids, are a class of natural products consisting of five-carbon isopentenyl diphosphate or its isomer dimethylallyl diphosphate as the structural unit [11]. In recent years, the new compounds isolated from AOF are mainly terpenoids, among which sesquiterpenoids are the most prevalent. As shown in Table 1, terpene compounds’ names and molecular formulas isolated from AOF are listed, and their corresponding structures are also depicted in Figure 1.

Wang et al. isolated 1*β*,4*β*,7*β*-trihydroxyeudesmane (**1**) and bullatantriol (**2**) from AOF by using silica gel column chromatography, MCI column chromatography, Sephadex LH-20 gel column chromatography, and the semi-preparative liquid method [12]. Thapa P et al. isolated 7*α*-hydroperoxy eudesma-3,11-diene-2-one (**3**), 7*β*-hydroperoxy eudesma-3,11-diene-2-one (**4**), and 3*α*-hydroxynootkatone (**5**) from MeOH extracts of AOF [13]. The compounds, oxyphylloneside A (**6**), oxyphylloneside B (**7**), carvacrol 2-*O*-*β*-glucopyranoside (**8**), thymoquinol 2-*O*-*β*-glucopyranoside (**9**) [14], (2*R*,5*R*,7*R*,10*S*)-2,7-dihydroxyl-eudesmane-3(4),11(12)-diene (**10**), *α*-rotunol (**11**), (1*S*,4*S*,5*R*,7*S*)-1-hydroxyl-eremophilane-9(10),11(12)-diene-8-one (**12**), cyperusol A1 (**13**), and (6*R*,9*S*,10*S*)-10-hydroxyl-11,12,13-trinor-cadinane-4(5)-ene-3-one (**14**) [15] were isolated by using the methods of MCI gel, Sephadex LH-20, silica gel, semi-preparative HPLC, and so on. Dong et al. isolated and characterized 17 eudesmane sesquiterpenoids from AOF, including (4*R*,5*R*,7*R*,10*R*)-12-nor-eudesma-4,5-epoxy-3-one (**15**), (3*S*,4*S*,5*R*,7*R*,10*S*)-eudesma-4,5-epoxy-11-en-3,7-diol (**16**), (4*S*,5*R*,10*S*)-12-nor-eudesma-6-en-4,5-diol-11-one (**17**), (3*S*,4*S*,5*R*,10*R*)-12-nor-eudesma-3,4-epoxy-6-en-5-ol-11-one (**18**), (3*R*,4*S*,10*R*)-11,12,13-trinor-eudesma-3,4-epoxy-5-en-7-one (**19**), (3*R*,4*S*,10*S*)-11,12,13-trinor-eudesma-3,4-epoxy-5-en-7-one (**20**), (6*S*,7*S*,10*R*)-eudesma-6,7-epoxy-4-en-3-one (**21**), (5*R*,7*R*,10*S*,11*S*)-eudesma-5,11-epoxy-3-en-12-ol (**22**), (5*S*,7*R*,10*S*)-eudesma-3-en-7,11,12-triol (**23**), (5*R*,7*R*,10*S*)-eudesma-4(15)-en-7,11,12-triol (**24**), (5*R*,7*S*,10*S*)-eudesma-4(15),11-dien-7-ol (**25**), (2*R*,5*R*,7*S*,10*S*)-eudesma-4,11-dien-2-ol (**26**), (4*R*,5*R*,7*R*,10*R*)-eudesma-11-en-5-ol-1-one (**27**), epialpiniol (**28**), alpinoxyphllaone A (**29**), alpinoxyphllaone B (**30**), and neoxyphyllanene (**31**) [16]. Qiu et al. used the liquid chromatography–mass spectrometry (LC-MS) method to separate oxyphylin A (**32**), oxyphylin B (**33**), oxyphylin C (**34**), oxyphylin D (**35**), oxyphylin E (**36**), oxyphylin F (**37**), oxyphylin G (**38**), oxyphylin H (**39**), oxyphylin I (**40**), and oxyphylin J (**41**) from AOF [17].

The extracts of AOF are rich in terpenoids. Bai et al. isolated (4S*,7S*,9R*)-14-nor-5(10),11(12)-dien-9-ol-1-one-eudesma (**42**) and cyperusol A4 (**43**) from EtOH extracts of AOF [18]. Dong et al. used a bioassay-guided method to isolate and purify ten sesquiterpenoids compounds from the crude extracts of AOF, obtaining alpinoxyphyllone C (**44**), (3*S*,4*S*,5*R*,7*R*)-eremophila-3,4-epoxy-1(10),11-dien-2-one (**45**), (3*R*,4*R*,5*R*,7*R*)- eremophila-3,4-epoxy-1(10), 11-dien-2-one (**46**), (4*R*,5*S*,7*S*,9*R*)-eremophila-1(10),11-dien-9-ol (**47**), (2*R*,4*R*,5*S*,7*S*,9*R*)-eremophila-1(10),11-dien-2,9-diol (**48**), (1*R*,2*R*,4*R*,5*S*,7*R*)-eremophila-9,11-dien-1,2-diol (**49**), oxyspirone J (**50**), oxyspirone K (**51**), (±)oxyspirone I (**52**), oxyspirone A (**53**), and oxyspirone B (**54**) [19]. The compounds alpinoxyphyllol A (**55**), alpinoxyphyllol B (**56**), 2-*O*-ethyl-*β*-nootkatol (**57**), 11-Hydroxyisohanalpinone (**58**), 6*S*-Oxyphyllenone H (**59**), 6*R*-Oxyphyllenone H (**60**) [20], oxyphylleudne J (**61**), oxyphyllerene D (**62**), and oxyphyllerene E (**63**) [21] were isolated from the EtOH extracts of AOF.

### 2.2. Flavonoids

**Figure 2:**
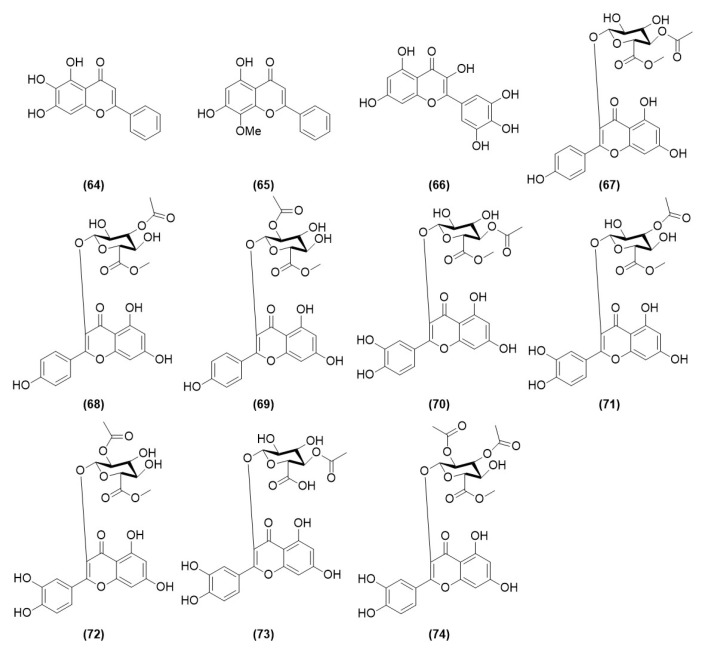
Structures of flavonoid compounds isolated from AOF.

Flavonoids are important indicators for evaluating the quality of *Alpinia oxyphylla* Miq and are also one of the main active chemical components in AOF [22]. Shi first isolated three flavonoids from the ethyl acetate extracts of AOF, namely, baicalein (**64**), wogonin (**65**), and myricetin (**66**) [23]. Zhu et al. isolated eight acetylated flavonol glucuronides from AOF by using preparative high-performance liquid chromatography and semi-preparative high-performance liquid chromatography, namely, Oxyphyllvinide A (**67**), Oxyphyllvinide B (**68**), Oxyphyllvinide C (**69**), Oxyphyllvinide D (**70**), Oxyphyllvinide E (**71**), Oxyphyllvinide F (**72**), Oxyphyllvinide G (**73**), and Oxyphyllvinide H (**74**) [24], as shown in Table 2 and Figure 2.

### 2.3. Diarylheptanoids

**Figure 3:**
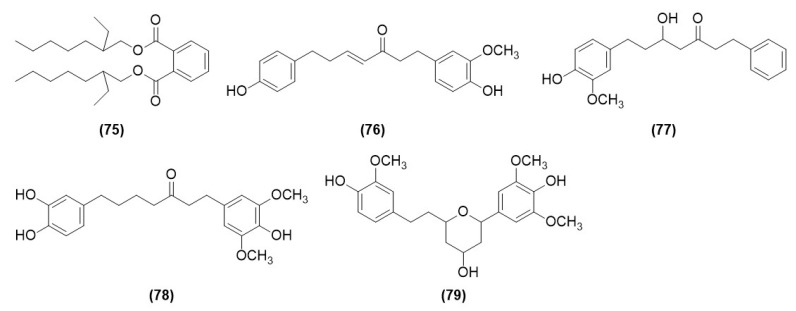
Structures of diarylheptanoid compounds isolated from AOF.

Diarylheptanoids, with a seven-carbon backbone and two phenyl rings at the 1- and 7-positions, are a family of secondary metabolites from plants [25]. Wang first isolated five chemical compounds of diarylheptanoids from the ethyl acetate parts of AOF, namely, 1, 2-benzenedicarboxylic acid (**75**), (*E*)-1-(4-hydroxy-3-methoxy-phenyl)-7-(4-hydroxy-phenyl)-hept-4en-3-one (**76**), 5-hydroxy-7-(4″-hydroxy-3″-methoxyphenyl)-1-phenyl-3-heptanone (**77**), dihydrogingerenone B (**78**), and 1,5-epoxy-3-hydroxy-1-(4-hydroxy-3,5-dimethoxyphenyl)-7-(4-hydroxy-3-methoxyphenyl)heptane (**79**) [12], as shown in Table 3 and Figure 3.

### 2.4. Other Components

**Figure 4:**
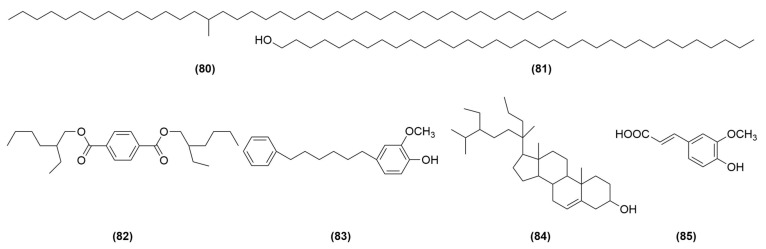
Structures of other compounds isolated from AOF.

As shown in Table 4 and Figure 4, alkanes, fats, esters, sterols, phenolic acids, and other compounds were also isolated from AOF in related studies. Wang et al. isolated and purified the compounds 15-methyl-tetracontane (**80**) and 1-tetratriacontanol (**81**) from AOF by using silica gel column chromatography, MCI column chromatography, Sephadex LH-20 gel column chromatography, and the semi-preparative liquid method [12]. Qiu et al. isolated bis-(2-ethylhexyl) terephthalate (**82**) from the ethyl acetate fraction of 90% EtOH extracts of AOF by silica gel, MCI, RP-18, Sephadex LH-20, TLC, and semi-preparative HPLC column chromatography [15]. Feng et al. isolate 3-methoxy-4-Hydroxy-diphenylhexane (**83**) and 20-propyl-β-sitosterol (**84**) from AOF [26]. Li et al. isolated and purified ferulic acid (**85**) from AOF by using silica gel column, MCI column, polyamide column, Sephadex LH-20 gel column, and semi-prepared liquid chromatography [27].

## 3. Pharmacology

In recent years, rich research results have been achieved in the pharmacology of AOF, including neuroprotection, regulation of metabolic disorders; antioxidant, antiapoptotic, anti-inflammatory, antidiabetic, antihyperuricemic, and antiaging properties; urinary shrinkage, immunomodulation; antitumor, renoprotective, hepatoprotective, antiasthmatic effects, etc. Table 5 lists the mechanism of pharmacological action of AOF.

### 3.1. Effects of AOF on Neurological Disorders

In recent years, some in vivo/in vitro research studies showed that the extract of AOF has the potential to treat neurological diseases by promoting neuroprotection [28,29,30], regulating metabolic disorders [31,32,33], having antioxidant, antiapoptotic [34,35,36,37,38,39], and anti-neuroinflammatory [40,41,42,43,44,45] properties, promoting nerve repair [46], etc.

#### 3.1.1. Neuroprotective Effects

Qiu et al. reported that terpenes extracted from AOF had neuroprotective effects on H_2_O_2_-induced SH-SY5Y cells. They isolated a total of 35 sesquiterpenoids, among which there were 9 compounds with significant neuroprotective effects [17]. Xiao et al. used the hCMEC/D3-HA1800 cell coculture model to validate the blood–brain barrier permeability of four active constituents derived from AOF, namely, protocatechuic acid, chrysin, tectochrysin, and nootkatone [47]. Xu et al. certified that chrysin was the main active substance in AOF to promote the transformation of microglia from the M1 to the M2 phenotype; in addition, triggering receptor expressed on myeloid cells-2 (TREM2) played a crucial role in promoting the polarization of the M2 phenotype. The anti-neuroinflammatory mechanism of chrysin might be related to the phosphoinositide 3-kinase (PI3K)/protein kinase B (AKT)/glycogen synthase kinase 3β (GSK3β) and brain-derived neurotrophic factor (BDNF)/tropomyosin receptor kinase B (TrkB)/toll-like receptors 4 (TLR4) signaling pathways [48]. Zeng et al. identified 49 active chemicals in AOF based on ultra-high-performance liquid chromatography with triple quadrupole mass spectrometry data and Lipinski’s rule of five, and 26 of these compounds targeted 168 key molecules for the treatment of neurodegenerative dementia. In addition, rhamnocitrin, rhamnetin, yakuchinone A, chrysin, dibutyl phthalat, butyl-*β*-*D*-fructopyranoside, oxyphyllacinol, oxyhylladiketone, 5-hydroxy-1,7-bis(4-hydroxy-3-methoxy-phenyl)heptan-3-one (5-HYD), and yakuchinone B were identified as polytargets of AO. hydroxy-1,7-bis (4-hydroxy-3-methoxyphenyl) heptan-3-one (5-HYD) and yakuchinone B were identified as key phytochemicals in the multitargeted modulation of neurodegenerative dementia pathogenesis in AOF [49]. Xu et al. showed that terpenes were the main constituents of AOF extracts and had neuroprotective effects [28]. Duan et al. found that the organic extracts of AOF had a protective effect against neurological damage caused by H2O2-induced apoptosis in PC12 cells [29]. Thapa et al. isolated eudesmane sesquiterpenes and eremophilane sesquiterpene from AOF, which were shown to be resistant to tertiary butyl peroxide-induced oxidative stress [13]. The investigation by Xu et al. demonstrated that chrysin had neuroprotective effects against glutamate-induced oxidative stress in PC12 cells [30].

In conclusion, AOF has good neuroprotective effects. However, the related studies are not sufficient and are mostly limited to the cellular level, and their mechanisms are not deeply studied. In addition, a variety of new compounds have been extracted and isolated from AOF in recent years, and in-depth research studies on the pharmacology and action mechanisms of these new compounds are conducive to the development and utilization of AOF. At present, the efficiency of drug development for central nervous system diseases is much lower than other diseases due to the poor efficiency of most drugs in crossing the blood–brain barrier [50]. It has been found that the main active ingredients of AOF can pass the blood–brain barrier, so in-depth research studies of the mechanism of the active ingredients of AOF passing the blood–brain barrier is conducive to the development of drugs for nervous system diseases.

#### 3.1.2. Treatment of Alzheimer’s Disease

Alzheimer’s disease (AD), a progressive neurodegenerative disease, can affect cognition and behavior, and it impacts the life quality of patients and their families [51]. Therefore, it is particularly important to seek effective ways to treat AD.

##### Regulation of Metabolic Disorders

It was found that several components in the total extracts of AOF could synergistically regulate metabolic disorders during the occurrence of AD, mainly involving amino acid metabolism, lipid metabolism, energy metabolism, etc. AOF might regulate metabolic disorders in AD by acting on protein targets such as fatty-acid amide hydrolase (FAAH), peroxisome proliferator-activated receptor gamma (PPARG), acetylcholinesterase (ACHE), cholinesterase (BCHE), and alcohol dehydrogenase 1C (ADH1C) through 17 pharmacological substances such as nootkatone, yizhiketone a, yizhiketone b, izalpinin, and chrysin, alleviating AD pathological progression and improving memory capacity [31]. Sun et al. reported that AOF could modulate metabolites in brain and plasma of model rats, which were mainly involved in the metabolism of amino acids, lipids, and energy [32]. Zhou et al. set up a metabolomic approach for bile acids (bas) to determine the levels of bas in different groups. AOF regulated the levels of cholic acid, chenodeoxycholic acid (CDCA), taurocholic acid (TCA), glycoconjugate cholic acid (GCA), taurochenodeoxycholic acid (TCDCA), glycoursodeoxycholic acid (GCDCA), deoxycholic acid (DCA), lithocholic acid (LCA), taurodeoxycholic acid (TDCA), taurolithocholic acid (TLCA), glycolithocholic acid (GLCA), etc., and the results suggested that the extracts of AOF might ameliorate the symptoms of AD in mice by regulating bas metabolism [33].

##### Antioxidant and Antiapoptotic Activity

The mechanisms for the treatment of AD with AOF are complex, involving multiple components, targets, and pathways. Li et al. found that adrenergic receptor alpha-1B (ADRA1B), muscarinic acetylcholine receptor M1 (CHRM1), ACHE, and monoamine oxidase B (MAOB) might be the important targets for the treatment of AD, and they may synergize to treat AD by regulating calcium balance, cholinergic balance, and phosphorylation. Four components, valencene, nootkatene, (+)-delta-cadinene, and caryophyllene oxide, were the potential active components of volatile oil from AO in the treatment of AD [52]. It was confirmed that the volatile oil from AO had a certain improvement effect on mice with scopolamine-induced learning and memory impairment. On the one hand, the volatile oil from AO might improve the body’s antioxidant ability by regulating the activity of acetylcholine (ACH) synthase and catabolase in the central nervous system. On the other hand, the volatile oil from AO regulated neuronal apoptosis-related factor expression by modulating the expression of BDNF, p-Extra Cellular-signal Regulated Kinase1/2 (p-ERK1/2), and p-Protein Kinase B 473 (p-AKT473) [34]. He et al. demonstrated that nootkatone had a protective effect on neurons, which was related to antioxidant and anti-ACHE activity. Nootkatone improved hippocampal histopathological changes by reducing the levels of amyloid beta (Aβ), malondialdehyde (MDA), and ACHE in the mouse brain and increasing the glutathione peroxidase (GSH-Px) levels, thus improving hippocampal histopathological changes [35]. Li et al. demonstrated that AOF might inhibit oxidative stress-induced apoptosis through the PI3K/Akt pathway in the treatment of AD [36]. He et al. found that tectochrysin had a neuroprotective effect, which attenuated learning and memory deficits in AD mice by inhibiting oxidative stress, the activity of total cholinesterase (TChE), and the accumulation of Aβ_1-42_ in the brain [37]. Bian et al. certified that oxyphylla A ameliorated cognitive deficits and reduced the expression levels of amyloid precursor protein (APP) and Aβ in AD mice, which exerted its antioxidant effects by the Akt-GSK3β and erythroid-derived 2-related factor 2 (Nrf2)- Keleh-like ECH-associated protein (Keap1)-heme oxygenase-1 (HO-1) pathways [38]. It was certified that AOF might exert its antiapoptotic effects via the caspase9–caspase3 pathway and was not dependent on caspase 8 [39].

##### Anti-Neuroinflammatory Activity

Wang et al. showed that nootkatone could ameliorate lipopolysaccharide (LPS)-induced learning and memory deficits in mice, which was associated with its attenuation of neuroinflammatory responses, and nootkatone significantly reduced the expression of interleukin 1β (IL-1β), interleukin 6 (IL-6), tumor necrosis factor (TNF-α), nuclear factor-kappa B (NF-κB) p65, and nucleotide-binding domain-(NOD-)-like receptor protein 3 (NLRP3) in the hippocampus of mice [40]. Yan et al. found that AOF improved cognitive deficits in the LPS-induced AD model through an anti-neuroinflammatory effect, which reduced the levels of TNF-α, IL-6, and IL-1β via the PI3K/AKT/NF-κB signaling pathway [41]. It was confirmed that the compound 2β-hydroxy-delta-cadinol (HOC) extracted from AOF had neuroprotective effects on Aβ-induced AD model mice and that HOC attenuated Aβ_1-42_-induced caspase-3 activation by inhibiting Aβ_1-42_-induced ROS production, which was able to inhibit caspase-3 activity [42]. Research has found that AOF had anti-neuroinflammatory effects, which could significantly inhibit the activation of the NF-κB pathway in the hippocampus of mouse models [45]. Wang et al. found that AOF might play a significant role in improving AD by acting on targets such as amyloid precursor protein (APP) and transcription factor AP-1 (JUN) via the combined action of active ingredients such as chrysin, stigmasterol, and protocatechuic acid, and the activated astrocytes can promote the expression of the targets in the pathway of advanced glycation end product (AGE)-receptor for AGE (RAGE) or interleukin 17 (IL-17) [43]. Yin et al. found that the serum containing AOF had no toxic effects on cells and effectively reduced the levels of NO, TNF-α, IL-1β, and NF-κBp65 produced by LPS-stimulated microglial cells, and its mechanism of action might be correlated with the NF-κB signaling pathway [44]. Shi et al. found that a novel acidic polysaccharide (AOP70-2-1) might be the active ingredient in the anti-neuroinflammatory activity of the crude polysaccharide of AOF, which obviously decreases the levels of IL-6, TNF-α, and NO produced by LPS-stimulated microglia [45].

**Figure 5:**
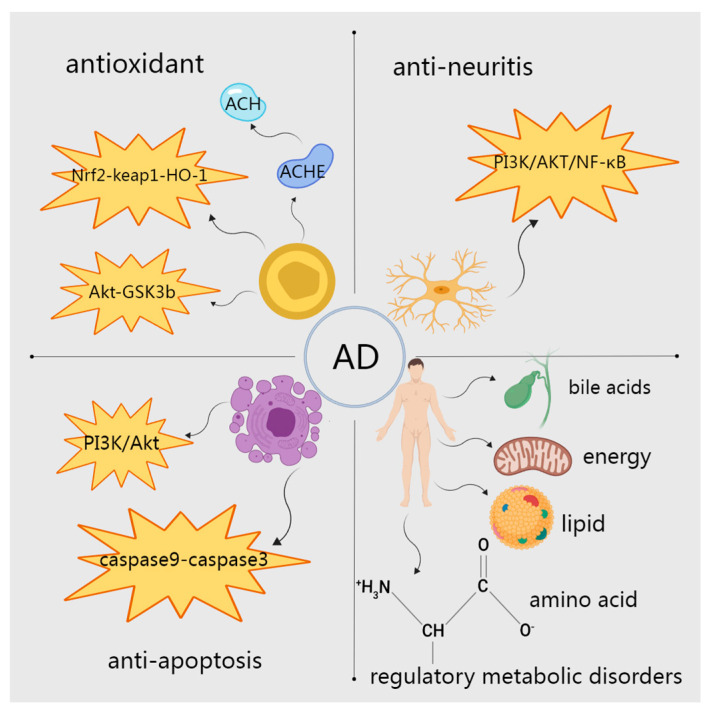
Mechanisms of AOF in treating AD.

To sum up, the main aspects of AOF in treating AD are related to the regulation of metabolic disorders and anti-inflammatory, antioxidant, and antiapoptotic activities (Figure 5), and there are more of the anti-inflammatory, antioxidant, and antiapoptotic aspects of AOF. In recent years, there has been a gradual increase in research on the regulation of metabolic disorders in the treatment of AD, and it is expected to become a research hotspot in the regulation of amino acid metabolism, lipid metabolism, energy metabolism, bile metabolism, and so on.

#### 3.1.3. Treatment of Stroke

As one of the most common neurologic diseases, stroke is a leading cause of death and long-term disability [53]. It has a serious impact not only on patients but also on their families, caregivers, businesses, and social networks [54]. Therefore, it is particularly important to study effective drugs for the treatment of stroke. Shi demonstrated that chrysin could play a neuroprotective role in cerebral ischemia–reperfusion injury by upregulating the expression of brain-derived neurotrophic factor (BDNF) and nerve growth factor (NGF) [23]. Tsai et al. found that the protective effect of AOF on the nerves of cerebral ischemia–reperfusion injury was related to the mechanism of mitochondrial apoptosis; the extracts of AOF inhibited the translocation of bcl-2-associated X (Bax) to the outer membrane of mitochondria by the modulation of the p38 mitogen-activated protein kinases (p38 MAPK)/90-kDa ribosomal S6 kinase (p90RSK) and c-Jun N-terminal kinase (JNK)/cathepsin B signaling pathways, which in turn preserved the mitochondria’s integrity [55]. Cheng et al. showed that the extracts of AOF ameliorated cerebral infarction in model rats by a mechanism concerned with the downregulation of JNK-mediated TLR4/tumor necrosis factor receptor-associated factor 3-interacting JNK-activating modulator (T3JAM) and ask1-associated inflammatory signaling pathways [56]. He et al. found that AOF was a drug that could promote hippocampal nerve regeneration and improve cognitive deficits after stroke, and its mechanism might be related to its activation of the BDNF/TrkB/AKT signaling pathway [46]. In conclusion, the treatment of stroke with AOF is related to its neuroprotective, neuroinflammatory, anti-inflammatory, and antiapoptotic effects.

#### 3.1.4. Others

Parkinson’s disease, epilepsy, and depression are neurological disorders that affect patients and their families and bring burdens to society [57,58,59]. Therefore, researching safe and effective therapeutic drugs is particularly important. Chen et al. found that oxyphylla A enantiomers isolated from AOF were neuroprotective against 1-methyl-4-phenyl-1,2,3,6-tetra-hydropyridine-induced neuroprotection in a zebrafish model of parkinsonism [7]. Zhou et al. demonstrated that oxyphylla A had the potential as an anti-Parkinson’s candidate, confirming that oxyphylla A had a protective effect on A53T α-synuclein(α-Syn)-induced neurotoxicity in both in vivo and in vitro experiments. The compound promoted A53T-α-syn degradation by activating the PKA/Akt/mechanistic target of rap (mTOR)/proteasome subunit beta type-8 (PSMB8) signaling pathway [60]. It was confirmed that the alcoholic extracts of AOF inhibited oxidative stress and apoptosis in the hippocampus of rats, and it reduced seizures by increasing the superoxide dismutase (SOD) content, decreasing the MDA content, ameliorating the dysregulated state between oxidative/antioxidant systems, and attenuating the damage of hippocampal neurons in epileptic rats [61]. Wu et al. certified that the volatile oil from AO had an antidepressant effect, the mechanism of which was to attenuate the depressive behavior of cums-induced mice by inhibiting the TLR4-mediated myeloid differentiation primary response protein (MyD88)/NF-κb signaling pathway and to reduce inflammatory factors such as IL-6, TNF-α, and IL-1β to improve neuroinflammation [62].

To summarize, AOF has neuroprotective effects and can treat neurological disorders such as AD, stroke, Parkinson’s, epilepsy, and depression. AOF treats neurological diseases by regulating metabolic disorders, having antioxidant, antiapoptotic, and anti-inflammatory properties, promoting nerve repair, and so on. Recent studies have shown that AOF can treat AD by regulating metabolic disorders such as those relating to amino acids, lipids, energy, and bile, but its mechanism needs further investigation. Furthermore, it remains to be explored by scholars whether the mechanism of AOF in treating other neurological diseases can be studied from the perspective of regulating metabolic disorders.

### 3.2. Hypoglycemic Effects

#### 3.2.1. Diabetes

Studies have certified that AOF contains a variety of sesquiterpenoids with hypoglycemic effects, which mainly exert hypoglycemic effects by stimulating the secretion of glucagon-like peptide-1 (GLP-1) antibody and inhibiting α-glucosidase and protein tyrosine phosphatase 1B (PTP1B) [20]. Xie et al. found that the extracts of AOF reduced the blood glucose level of model mice by regulating the composition of intestinal microbiota, which increased ratios of bacteroidetes to firmicutes in intestinal microorganisms and regulated the intestinal flora [63].

#### 3.2.2. Diabetic Nephropathy

Zong et al. found that it had a protective effect on the kidneys and significant efficacy in the treatment of diabetic nephropathy (DN) in the early stages of the disease and that a decoction of AOF could effectively reduce blood glucose as well as reduce the excretion of urinary microalbumin [64]. It was reported that the mechanism of the protective effect of AOF in DN mice might be correlated with the regulation of lipid metabolism, including sphingolipin, phosphatidylcholine, lysophosphatidylcholine, and phosphatidylethanolamine [65]. The study demonstrated that AOF could reduce blood sugar, urea nitrogen, urinary albumin, and urinary creatinine, inhibit oxidative stress, and improve renal pathological status in mice with DN, and it could regulate the metabolomics and intestinal tract [66]. Jia et al. certified that the therapeutic effects of crude AOF (CAOF) and salt AOF (SAOF) on renal injury might be related to their effects on increasing intestinal bacterial diversity; CAOF and SAOF reduced the level of transforming growth factor-β1 (TGF-β1) and attenuated DN renal fibrosis. Moreover, both increased the intestinal bacterial diversity in DN rats and increased the level of intestinal bacterial metabolite short-chain fatty acid (SCFA) levels [67].

In summary, AOF had hypoglycemic and renal protective effects, which were mainly exerted by regulating intestinal flora, regulating metabolism and having antioxidant and antifibrotic effects. However, the above research studies are not in-depth, and the specific mechanism of hypoglycemic and renal protection of AOF has not yet been clarified and still needs to be studied in the future.

### 3.3. Antihyperuricemia

Sung et al. showed that allopurinol combined with the extracts of AOF could reduce serum concentration of uric acid in model rats, which involved a dual mechanism. On the one hand, it increased uric acid (UA) excretion by regulating urate transporter 1 (URAT1) and organic anime transporter 1 (OAT 1). On the other hand, it reduced UA production by inhibiting xanthine oxidase (XOD) [68]. Lee et al. certified that the extracts of AOF possessed the functions of antihyperuricemia and promoting uric acid excretion, and its mechanism of action was related to the inhibition of UA reabsorption by enhancing the renal secretion of UA [69].

### 3.4. Antiaging

Yang et al. reported that the extracts of AOF could improve caenorhabditis elegans’s antioxidant and high-temperature resistance abilities by promoting the activity of antioxidant enzymes and the expression level of antioxidant genes, thus delaying caenorhabditis elegans’s senescence and increasing its lifespan [70]. Xiao et al. showed that AOF could extend caenorhabditis elegans’s lifespan and healthspan by improving the activity of antioxidant enzymes, which involves regulating the gene expression of the insulin/insulin-like growth factor-1 (IGF-1) and SKN-1 signaling pathways [71]. Chang et al. confirmed that the combination of the extracts from AOF and adipose-derived mesenchymal stem cells (ADMSCs) effectively inhibited mitochondria-mediated cardiac apoptosis and improved cardiac function in aging rats, and the combination could reduce the expression levels of aging-related markers P21 and β-galactosidase and downregulate the expression levels of mitochondria-mediated apoptosis markers caspase-3 and cyt c. In addition, the expression of survival and longevity markers pAKT and silent mating type information regulator 1 (SIRT1) was increased in the ADMSC group treated with AOF extracts, suggesting that AOF might increase the specificity and stability of ADMSCs to recover from aging-induced cardiac damage [72]. Lin et al. certified that ADMSCs and the extracts of AOF synergistically ameliorated the complications associated with the aging rat heart, and the mechanism may be related to the activation of the Nrf2 pathway to partially alleviate oxidative stress and inhibit inflammation [73]. Lu et al. found that tectochrysin had an antiaging function, which could obviously prolong the lifespan of the caenorhabditis elegans, promote the caenorhabditis elegans’s resistance to high-temperature stress and infection, delay the decline of caenorhabditis elegans motility, and reduce the Aβ_1-42_-induced toxicity [74]. Additionally, the antiaging effect of tetracycline was related to FOXO/DAF-16 and heat shock factor 1 (HSF-1) [74]. The investigation by Chang et al. certified that the extracts of AOF could ameliorate D-galactose-induced collagen deposition, inhibit the expression of TGF-β1, and downregulate matrix metalloproteinases-2 (MMP-2) and matrix metalloproteinase-9 (MMP-9) in an aged myocardial injury model rat, thereby attenuating aging-induced cardiac fibrosis [75]. It was found that AOF had the efficacy of improving the decline of male reproductive function caused by aging, and it could improve sperm production, increase the production and action of testosterone, and attenuate oxidative stress [76].

All in all, AOF has an antiaging function, which is related to antioxidant activity, antiapoptosis, antifibrosis of the heart, and promotion of sperm production. The antiaging mechanism of AOF is not deeply studied, and further research studies are needed in the future.

### 3.5. Antidiuresis

Studies have shown that AOF reduced collagen synthesis via the transforming growth factor β 1 (TGFβ1)-SMAD family member 3 (SMAD 3) signaling pathway to inhibit bladder fibrosis caused by overexpression of collagen. What is more, AOF effectively inhibited the G protein (Gq)- phospholipase C beta 1 (PLCβ1) calcium signaling pathway, which reduced the expression of myosin light chain kinase and myosin light chain, thus reducing bladder contraction frequency [77]. Su et al. reported that intermittent hypoxia (IH) regulated proliferation and apoptosis of urothelial cells via P2X3 bladder nerve receptors, and that medium (50 mg/L) and high (100 mg/L) doses of AOF altered calcium channels and protected against IH-induced cell damage [78]. Feng et al. showed that the 75% ethyl acetate extracts of 75% ethanol extract AOF had a significant inhibitory effect on urination in mice, and this fraction obtain two compounds with antidiuretic effects [26]. Han et al. demonstrated that polysaccharide-form AOF could significantly improve the symptoms of urinary incontinence and the water–electrolyte balance; it also increased the content of aldosterone (ALD) and antidiuretic hormone (ADH) and promoted the expression of the β3-AR-cAMP signaling pathway in the model rats. Moreover, polysaccharide-form AOF increased the indices of spleen, thymus, and adrenal, suggesting that the treatment of urinary incontinence by polysaccharide-form AOF may be correlated with its immune-modulating effects [79]. Su et al. confirmed that AOF improved anischuria by inhibiting oxidative stress and modulating the receptor expression of purinergic P2X3, muscarinic M3, and β3 adrenergic [80].

### 3.6. Immune Regulation

Liu found that AOF increased the phagocytic index and phagocytosis rate of rat macrophages and increased the natural killer (NK) cell activity, suggesting that it has an enhancing effect on both humoral and cellular immunity [81]. Tian et al. reported that AOF could improve the immune function of mice by increasing the indices of thymus and spleen and the phagocytic index α. In addition, AOF also stimulated the differentiation and proliferation of immunoreactive cells through the proliferation of splenic lymphocytes induced by Con A, thus improving the cellular immunity of the organism [82]. Yang et al. showed that AOF polysaccharide (AOFP) could promote the proliferation of mouse splenic lymphocytes, in which AOFP1 promoted the secretion of interleukin 2 (IL-2), interleukin 4 (IL-4), IL-6, Interferon-γ (IFN-γ), and the production of a specific antibody (sIgG) in a concentration-dependent manner, suggesting that AOFP1 had an enhancement effect on both the Th-1 and Th-2 types of immune responses and specific immune responses [5]. All three AOFPs could promote the activation of RAW264.7 cells, with AOFP2 having the strongest effect, and AOFP2 polarized RAW264.7 cells toward the M1 and M2 phenotypes and enhanced the th2-type immune response by promoting the secretion of factors, such as nitric oxide (NO), IL-6, interleukin 10 (IL-10), TNF-α, inducible nitric oxide synthase (iNOS), TGF-β, etc. [83].

In summary, AOF has the effect of regulating immunity, and can regulate the immune function by regulating two immune organs, namely, the thymus and the spleen. The above studies on the effect of AOF on immune organs are not in-depth, and there are no studies on immune organs such as bone marrow, tonsils, and mucous membrane-associated lymphoid tissues, which are expected to become hot spots for research in the future. In addition, the above studies only studied lymphocytes, phagocytes, NK cells, and T cells, but the studies were not in-depth. In the future, the regulatory effect of AOF on dendritic cells, eosinophils, basophils, and other immune cells can be further studied. Moreover, the research on immunoglobulin, complement, cellular molecules, and other immune molecules of AOF is still missing, and this area may become a hot spot of research in the future. At present, it has been shown that AOF has a therapeutic effect on autoimmune encephalomyelitis; whether it has a therapeutic effect on other autoimmune diseases is still to be further studied.

### 3.7. Anti-Inflammatory Activity

It was confirmed that compounds isolated from AOF could inhibit the LPS-stimulated secretion of inflammatory factors such as NO, TNF-α, and IL-6 from microglia. Among them, (3*S*,4*aS*,5*R*)-2,3,4,4a,5,6-hexahydro-4a,5-dimethyl-3-(1-methyl-ethenyl)-1,7-naphthalenedione can exert anti-inflammatory effects by downregulating the expression of pro-inflammatory biomarkers such as TNF-α, IL-6, iNOS, and cyclooxygenase-2 (COX-2) and inhibiting the phosphorylation of ERK1/2, JNK, and p38. [19]. Bai et al. isolated and identified four sesquiterpenoids from AOF, four compounds with anti-inflammatory activity that could reduce PGE2 production by LPS-stimulated RAW 264.7 macrophages [18]. Busayo et al. isolated two compounds, nootkatone and valencene, from the fruits and leaves of *Alpinia oxyphylla* Miquel, and both compounds inhibited production of NO by LPS-stimulated RaW264.7 cells [84]. In addition, nootkatone had tyrosinase inhibitory activity [84]. Dong et al. isolated a variety of sesquiterpenoids with anti-inflammatory effects from AOF, some of which inhibited production of NO and some of which inhibited the LPS-stimulated secretion of IL-6 and TNF-α in BV-2 cells [16]. Moreover, compound 1-oxo-5α,7αH-eudesm-3-en-15-al downregulated the protein expression of iNOS and COX-2 and significantly reduced the expression of IL-6, TNF-α, iNOS, and COX-2 mRNAs [16]. Yu et al. demonstrated the anti-inflammatory and analgesic effects of the extracts of AOF, which effectively inhibited three animal inflammation models, including carrageenan-induced rat paw edema, acetic acid-induced mice writhing, and arachidonic acid-induced mice ear edema. Furthermore, in vitro sulfated glycosaminoglycan (GAG) inhibition experiments showed that the extracts of AOF can be used for the treatment of osteoarthritis or related symptoms [85]. Jeon et al. certified that the extracts of AOF had an inhibitory effect on IL-1α and plasminogen-induced collagen degradation, which restored the balance between MMPs and TIMPs by decreasing the expression of MMP-3 and MMP-13 and increasing the expression of TMIP-1 [86]. Moreover, the AOF extracts reduced the production of PGE2 and NO and inhibited the inflammatory response [86]. The investigation by Lee et al. showed that the extracts of AOF could attenuate iodoacetate-induced osteoarthritis in rats, inhibit the production of pro-inflammatory cytokines such as IL-1β, IL-6, and LTB4, and inhibit the production of the bone turnover markers osteocalcin and deoxypyridinoline (DPD). In addition, the in vitro results suggested that the extracts of AOF had an anti-inflammatory effect on LPS-treated RAW264.7 cells, which reduced the expression level of IL-1β, IL-6, PGE2, and NO by inhibiting the MAPK signaling pathway [87].

In summary, AOF has anti-inflammatory and antiarthritic effects, but its mechanisms are not well studied, and further research studies are needed in the future. In addition, it has been shown that AOF has a therapeutic effect on osteoarthritis, but whether it has a therapeutic effect on rheumatoid arthritis, ankylosing spondylitis, gouty arthritis, and other arthritic diseases is yet to be further researched.

### 3.8. Antitumor Activity

It has been found that the ethyl acetate extracts of AOF could inhibit the activation of the IL-6/STAT3 pathway and the expression of Ki-67 and MMP2 proteins, which in turn reduced the proliferation, migration, and invasive ability of cholangiocarcinoma TFK-1 cell line, with dose-dependent effects [88]. Yoo et al. reported that nootkatone, an active extract of AOF, had an inhibitory effect on colorectal cancer cells; the mechanism of the antiproliferative activity of nootkatone might be related to the increase in the transcriptional expression of nonsteroidal anti-inflammatory drug-activated gene-1 (NAG-1) by EGR-1 and the degradation of cyclin D1 through a mechanism related to the proteasomal degradation pathway [89]. Hui et al. demonstrated that the petroleum ether extract of AOF significantly inhibited the growth of Hep3B, SMMC-7721, BEL-7402, and HepG2 cells in a concentration-dependent and time-dependent manner and that it induced apoptosis by increasing the expression of PTEN and decreasing the expression of p-AKT and PI3K, thereby inhibiting the growth of hepatocellular carcinoma [90].

### 3.9. Renal Protection

Dai et al. showed that nootkatone could markedly promote acute kidney injury induced by CCl_4_ exposure in model mice, and its mechanism might be related to the NAD(P)H oxidase-4 (NOX4), NF-κB, Nrf2/HO-1 pathway and mitochondrial apoptosis. Additionally, nootkatone partially inhibited production of NOX4 and MDA and increased the expression level of glutathione (GSH), catalase (CAT), glutathione peroxidase (GPX), and SOD, thus inhibiting oxidative stress. Furthermore, nootkatone could also exert antioxidant effects by upregulating the expression of Nrf2 and its downstream target HO-1 mRNA. In terms of anti-inflammatory effects, nootkatone reduced the expression levels of TNF-α, IL-6, IL-1 β, iNOS, and NO by inhibiting the NF-κB pathway; in terms of mitochondrial apoptosis, nootkatone can improve renal toxicity by reducing the expression levels of caspase-9 and caspase-3 to block mitochondrial apoptosis [91]. It was confirmed that the compounds oxyphyllvonide G, 3″-*O*-acetyl-*β*-*D*-glucuronide, 2″-*O*-acetyl-*β*-*D*-glucuronide, oxyphyllvonide H, quercetin 3-*O*-*β*-*D*- glucuronide, kaempferol 3-*O*-*β*-*D*-glucuronide, quercetin 6*-O*-*α*-*L*-rhamnosyl-*β*-*D*-glucoside, and genistein 7-*O*-*β*-*D*-glucoside had an inhibitory effect on TGF-β1-induced kidney proximal tubule cell renal fibrosis. Furthermore, oxyphyllvonide G, 3″-*O*-acetyl-*β*-*D*-Glucuronide, and kaempferol 3-*O*-*β*-*D*-glucuronide were superior to nootkatone in inhibiting fibronectin expression [24].

In summary, the compounds of AOF have nephroprotective effects related to antioxidant anti-inflammatory activity, antimitochondrial apoptosis, and antirenal fibrosis. In recent years, there have not been many studies on the nephroprotective effects of AOF, and its main related components are not well studied; in the future, in-depth studies on the mechanism of action of other active components of AOF are still needed.

### 3.10. Hepatoprotection

AOF exerts a protective function on the liver. Zhang et al. confirmed that nootkatone significantly ameliorated the disorders of liposugar metabolism and reduced the accumulation of fat in liver tissues in metabolic-associated fatty liver disease (MAFLD) mice, thereby ameliorating the liver injury [92]. And the mechanism might be related to the activation of the AMP-activated protein kinase (AMPK) pathway and the inhibition of the mitogen-activated protein kinase (MAPK) pathway by nootkatone [92]. Park et al. found that the extracts of AOF had antioxidant effects and protected against tert-butyl hydroperoxide-induced cytotoxicity of HepG2 and that eudesma-3,11-dien-2-one and yakuchinone A, chemical components of AOF, could exert antioxidant effects by activating the Nrf2/HO-1 pathway and scavenging free radicals. Thus, AOF may be used as a potent native antioxidant for preventing and treating oxidative stress-associated liver diseases [93].

### 3.11. Antiasthma

Fang et al. found that tectochrysin was protective against shrimp tropomyosin-induced allergic asthma in mice and that tectochrysin ameliorated allergic airway inflammation by inhibiting Th2 responses and oxidative stress [94].

**Table 5:** Mechanisms of pharmacological action of AOF.

Pharmacology	Diseases	Model	Pathways	Effects	References
Neuroprotection	/	In vitro	/	↓ ROS, MDA, NO	[17]
	/	In vitro	PI3K/AKT/GSK3β, BDNF/TrkB/TLR4	↓ Iba-1, NO, TNF-α, iNOS ↑ TREM2, Arg-1, IL-10	[48]
Regulation of metabolic disorders	AD	In vitro	/	↑ arginine, 2-hydroxy-2,4-pentadienoate, succinic semialdehyde, LPE, isocitrate, acetyl-l-carnitine, palmitoylcarnitine, oleoylcarnitine ↓ isoleucine, glutamine, LPC, arachidonic acid, eicosapentaenoic acid, docosapentaenoic acid, docosahexaenoic acid, adrenic acid	[32]
	AD	In vitro	/	↑ CA, CDCA ↓ TCA, GCA, TCDCA, GCDCA, LCA, TDCA, TLCA, GLCA	[33]
	AD	In vitro	/	↓ DCA	[32,33]
Antioxidant, antiapoptotic	AD	In vivo	/	↑ BDNF, ERK, CREB, BCL-2, p-ERK1/2, p-AKT473 ↓ BAX, cleaved caspase-3	[34]
	AD	In vivo	/	↓ Aβ, ACHE	[35]
	AD	In vivo	/	↓ TChE, ↑ SOD	[37]
	AD	In vivo	/	↓ MDA, ↑ GSH-Px	[35,37]
	AD	In vivo	PI3K/Akt	↑ SOD, CAT, GSH-Px, MMP, Bcl2 ↓ ROS, caspase-3, BAX	[36]
	AD	In vivo	Akt-GSK3b, Nrf2-Keap1-HO-1	↓ APP, Aβ	[38]
	AD	In vitro	NF-κB, caspase9- caspase3	↑ IKK-α, p65 ↓ IκB-α, p53	[39]
Anti-neuroinflammatory	AD	In vitro	/	↓ IL-1β, NFκB p65, NLRP3	[40]
		In vivo	/	↓ NO	[45]
		In vitro/in vivo	/	↓ TNF-α, IL-6,	[40,45]
		In vitro	PI3K/AKT/NF-κB	↓ TNF-α, IL-6, IL-1β	[41]
		In vitro	/	↓ ROS, caspase-3	[42]
		In vivo	NF-κB	↓ NO, TNF-α, IL-1β, NF-κBp65	[44]
Effects on other neurological disorders	Stroke	In vivo	/	↑ GalC, NGF, NSE, Nestin, Vimentin	[23]
	Stroke	In vivo	p38 MAPK/ p90rsk, JNK/Cathepsin B	↑ p-Bad, CREB, Bcl-2 (Bcl-xL)/Bax ↓ Bax, p53, cyto c, Smac/DIABLO, AIF, caspase	[55]
	Stroke	In vivo	TRAF3/T3JAM/JNK, JNK/NF-κ b, TLR4/T3JAM/JNK-, ASK1/JNK, TLR4/Iba1 (GFAP)/TRAF3/ T3JAM-, NF-κ b /ASK1, TLR4/JNK, ASK1/JNK	↓ iNOS, COX-2, TNF-α, IL-6	[56]
	Stroke	In vivo	BDNF/TrkB/AKT	↑ BDNF, TrkB, p-AKT	[46]
	PD	In vivo	PKA/Akt/ mTOR/PSMB8	↓ A53T-α-syn	[60]
	Epilepsy	In vivo	/	↑ SOD ↓ MDA	[61]
	Depression	In vitro	MyD88/NF-κ b	↓ IL-6, TNF-α, IL-1β	[62]
Reduction of blood sugar	Diabetes	In vitro	/	↓ α-glucosidase, PTP1B ↑ GLP-1	[20]
	Diabetes	In vivo	/	↑ ratio of bacteroidetes to firmicutes ↓ blood glucose	[63]
	DN	In vivo	/	↓ blood glucose	[64,66]
	DN	In vivo	/	↓ sphingolipin, phosphatidylcholine, lysophosphatidylcholine, phosphatidylethanolamine, urea, nitrogen, urinary creatinine, urinary albumin	[65,66]
	DN	In vivo	/	↓ urinary excretion of microalbumin	[64]
	DN	In vivo	/	↓ H2O2, MDA, Superoxide Anion ↑ CAT, T-GSH, SOD	[66]
	DN	In vivo	/	↓ TGF-β1, UP, BUN, TG, TC, LDL-C, Scr, BUN, LDL-C, abundance of proteobacteria ↑ SCFAs, HDLC	[67]
Antihyperuricemic	Hyperuricemia	In vivo	/	↓ GOT, BUN, IL-1β,	[68]
	Hyperuricemia	In vivo	/	↑ OAT1 ↓ URAT1, UA	[68,69]
Antiaging	/	In vitro	/	↑ CAT, SOD, DAF-16, SOD-3	[70]
	/	In vitro	insulin/IGF, SKN-1	↑ SOD, CAT, sod-3, gst-4, daf-16, skn-1 ↓ daf-2, age-1, ROS, MDA	[71]
	/	In vitro/in vivo	/	↓ P21, β-galactosidase, cyt c, caspase-3 ↑ pAKT, SIRT-1	[72]
	/	In vitro	Nrf2	↑ Nrf2, HO-1, SOD-1, IκBα, TIMP-1 ↓ Rac-1, Nox-2, IL-6, p-NF-κB, CTGF, MMPs	[73]
	/	In vitro	/	↑ DAF-16	[74]
	/	In vivo	/	↓ TGF-β1, MMP-2, MMP-9	[75]
	/	In vivo	/	↑ spermatids, testosterone ↓ 8-Hydroxydeoxyguanosine, Histone deacetylases 1, caspase-3	[76]
Antidiuretic	Overactive bladder	In vivo	TGFβ1-SMAD 3, Gq-PLCβ1 calcium	↓ TGF-β1, SMAD 3, Collagen, Gq, PLC β1, MLCK, MLC	[77]
	/	In vitro	/	↓ α-smooth muscle actin; ↑ apoptosis rate of human bladder detrusor cell	[78]
	/	In vivo	/	↓ urine volume	[26]
	/	In vivo	β3-AR-cAMP	↑ ALD, ADH, splenic coefficients, thymus coefficients, adrenal coefficients, AC, cAMP, PKA, β3-adrenoceptor mRNA	[79]
	/	In vivo	/	↓ MDA, P2X3, muscarinic M3 ↑ SOD, β3-adrenergic receptor	[80]
Immune regulation	/	In vivo	/	↑ phagocytosis rate, NK cells	[81]
	/	In vitro	/	↑ thymus index, spleen index	[82]
	/	In vitro/in vivo	/	↑ phagocytic index	[81,82]
	/	In vitro	/	↑ IL-2, IL-4, IL-6, IFN-γ, sIgG	[5]
	/	In vitro	/	↑ NO, IL-10, TNF-α, iNOS, TGF-β	[83]
Anti-inflammatory	/	In vitro	/	↓ TNF-α, IL-6, iNOS, COX-2	[16,19]
	/	In vitro	/	↓ PGE2	[18]
	/	In vitro	/	↓ NO	[19,84]
Antiarthritic	Osteoarthritis	In vivo	/	↓ GAG	[85]
	Osteoarthritis	In vivo	/	↓ MMP-3, MMP-13, PGE2, NO ↑ TMIP-1	[86]
	Osteoarthritis	In vitro/in vivo	MAPK	↓ IL-1β, IL-6, PGE2, NO	[87]
Antitumor	Bile duct cancer	In vitro	IL-6/STAT3	↓ IL-6, p-STAT3, MMP2, Ki-67	[88]
	Colorectal cancer	In vitro	/	↑ NAG-1 ↓ cyclin D1	[89]
	Liver cancer	In vitro/in vivo	/	↑ PTEN ↓ p-AKT, PI3K	[90]
Renal protection	-/	In vivo	NOX4, NF-κB, Nrf2/HO-1	↑ GSH, CAT, GPX, SOD ↓ NOX4, MDA, TNF-α, IL-6, IL-1β, aspase-9, caspase-3	[91]
	/	In vitro	/	↓ fibronectin	[24]
Hepatoprotection	Metabolic fatty liver disease	In vivo	MAPK	↓ IL-1β, IL-18, TNFα, IL-6, TG, HDL-c, p-ERK1/2, p-p38, p-JNK ↑ p-AMPKα	[92]
	/	In vitro	Nrf2/HO-1	↑ Nrf2, Ho1	[93]
Antiasthmatic	Asthma	In vivo	/	↓ IL-4, IL-5, IgE, CD200R ↑ CAT, GPX	[94]

(↑ means increase, ↓ means decrease).

## 4. Research on the Herb Pairs of AOF

An herb pair (HP) is concerned with two relatively fixed herbs in Chinese medicine that are commonly used in the clinical compounding of TCM [95], which can either promote each other to enhance a therapeutic effect or restrain each other to reduce toxic side effects. By studying the herb pairs of AOF and summarizing their potential compatibility mechanisms (Table 6), we aim to provide reference for further research on AOF.

### 4.1. AOF–Schisandra Chinensis

The investigation by Wang et al. showed that the AOF–Schisandra chinensis (AOSC) herb pair improved cognitive dysfunction in model mice by reducing the expression of β-secretase (BACE1), lowering the level of A β_1-42_, and controlling the excessive phosphorylation of tau protein. In addition, its ratio (AO:SC) of 1:2 could better exert neuroprotective effects (Table 6) [96]. It was confirmed that AOSC extracts significantly reversed Aβ_1-42_-induced behavioral cognitive deficits, which was correlated with its inhibition of the NF-κB pathway and antiapoptotic effects. What is more, the antiapoptotic effect of AOSC might be independent of caspase 8, which exerts its antiapoptotic effect by activating caspase 3 directly through caspase 9 (Table 6) [97]. Qi et al. found that AOSC ameliorated the cognitive deficits in the Aβ_1-42_-induced mouse model of AD and that AOSC reduced Aβ_1-42_ deposition and downregulated the hyperphosphorylation of tau proteins, possibly via the PI3K/Akt/Gsk-3β/CREB pathway, thereby reversing cognitive deficits and neurodegeneration in the mice (Table 6) [98]. Schisandrin and nootkatone, two core components, respectively, extracted from SC and AOF in combination had neuroprotective effects on the AD mouse model. On the one hand, schisandrin and nootkatone exerted neuroprotective effects by inhibiting the TLR4/NF-κB/NLRP3 inflammatory signaling pathway and reducing oxidative stress damage [99]; on the other hand, the pair exerted anti-inflammatory effects by stimulating the PI3K/AKT/Gsk-3β/mTOR pathway and decreasing the levels of inflammatory factors, such as TNF-α, IL-1β, IL-6, NF-κB, IKK, etc. Moreover, the decreased expression levels of cleaved-Caspase3 and LC3-II suggested that it had an inhibitory effect on apoptosis and autophagy (Table 6) [100]. Wang et al. found that AOSC lowered the levels of ACh and M1 receptors and ACHE activity, thereby improving cognitive impairment in model mice [101].

### 4.2. AOF–Acorus Tatarinowii Schott

Studies have shown that the essential oil of the AOF–Acorus tatarinowii schott herb pair (AOAT) could effectively improve the behavior of AD mice, reduce the phenomenon of hippocampal neuron deletion and nuclear consolidation in mice, increase the content of acetylcholine in the brain tissue, and reduce the content of γ-aminobutyric acid and glutamate in the brain tissue. Moreover, the therapeutic effect of the essential oil of AOAT in AD mice was related to energy metabolism, and it could significantly increase the level of glucose transporter 1 (GLUT-1) and inhibit insulin receptor substrate 1 (IRS-1) levels in brain tissue (Table 6) [102]. Li showed that volatile oils of AOAT had reparative and protective effects on human cerebral microvascular endothelial cells (hCMEC/D3) with Aβ25-35-induced injury, and the mechanism of action might involve the modulation of the cellular expression levels of proteins, such as RAGE, LRP1, GLUT1, GLUT3, and so on (Table 6) [103].

### 4.3. AOF–Lindera Aggregata

Studies have shown that the treatment of DN by the AOF–Lindera aggregata (AOLA) herb pair were related to the regulation of cellular autophagy [104], and its mechanism was to promote the autophagy of podocytes through the modulation of the PI3K/Akt/mTOR signaling pathway to improve the renal pathological damage, thus exerting a therapeutic effect for the treatment of DN (Table 6) [105]. Mao et al. demonstrated that both AOF and Lindera aggregata could inhibit high glucose-induced proliferation of glomerular thylakoid cells by altering the expression of miNA-21 in thylakoid cells and regulating the PTEN/PI3K/AKT signaling pathway, and the AOLA pair was better than that of the two herbs alone (Table 6) [106].

### 4.4. AOF–Other Herb Pairs

It was reported that the AOF–Foeniculum vulgare (AOFV) herb pair had an antidiarrheal effect on diarrhea of spleen–kidney Yang deficiency, and the mechanism of the antidiarrheal effect of the salt herb pair might be correlated with analgesia, regulation of energy metabolism, and immune function of the body (Table 6) [107].

Gao et al. found that 80% ethanol-eluted flavonoids of AOF–Amomi fructus (AOAF) as a mixture compounded at a ratio of 1:1 had a strong bacteriostatic effect, and its bacteriostatic effect was stronger than that of the single bacteriostatic effects of AOF and amomi fructus. Moreover, the mixture inhibited the transplanted tumors S180 and H22 in mice (Table 6) [108]. Protocatechuic acid and bornyl acetate are each a main active component of AOF and Amomi fructus, respectively; these were effective in okadaic acid (OA)-induced model cells of AD, and they reduced tau protein and lactate dehydrogenase (LDH) levels in the OA-induced AD cell model [109].

Zhang et al. demonstrated that the AOF–Schizonepeta tenuifolia (AOST) herb pair had an anti-inflammatory effect and alleviated allergic dermatitis in model mice by improving the Th1/Th2 balance and decreasing pro-inflammatory cytokines, thus alleviating the symptoms of the model mice (Table 6). Moreover, the effect of the pair on regulating immunity and anti-inflammation might be related to the gut–microbiome–skin axis [110].

To summarize, the studies of herb pairs mainly include AOSC, AOAT, AOLA, AOFV, AOAF, and AOST, with AOSC being the main type (Table 6). Diseases involved include AD, DN, diarrhea, allergic dermatitis, etc., with AD being the main type. AOSC pairs are effective in treating AD, and the study of its therapeutic mechanism is expected to be the focus of future research. In addition, most of the studies on drug pairs of AOF are on the mixed extracts of herb pairs. Clarifying the mechanism of action, main active ingredients, and ratio between single compounds of mixed extracts is beneficial for exploring the potential mechanisms of HPs, deepening research on AOF, and providing reference for clinical application.

**Table 6:** Action mechanisms of AOF pairs.

Herb Pairs/Components	Pharmacology	Diseases	Model	Pathways	Effects	References
AOSC	Neuroprotection	AD	In vitro/in vivo	/	↓ BACE1, Aβ_1–42_	[96]
	Neuroprotection	AD	In vitro	PI3K/Akt/Gsk-3β/CREB	↓ Aβ_1-42_	[98]
	Neuroprotection	AD	In vivo	/	↑ ACH, M1 ↓ ACHE	[101]
	Anti-inflammatory	AD	In vivo	NF-κB	↓ IKK-α, NF-κB, p53, Bad, Bax ↑ Bcl-2, Bcl-xl, i-κB-α	[97]
Schisandrin and nootkatone	Neuroprotection	AD	In vivo	TLR4/NF-κB/NLRP3	↓ TNF-α, IL-1β, IL-6mRNA, COX-2, iNOS, GSH-Px, MDA, TAOC, NO ↑ GST, T-AOC, SOD, GSH	[99]
	Anti-inflammatory, inhibition of apoptosis and autophagy	AD	In vitro/in vivo	PI3K/AKT/Gsk-3β/mTOR	↓ TNF-α, IL-1β, IL-6, NF-κB, IKK, cleaved-caspase3, LC3-Ⅱ	[100]
AOAT	Regulation of energy metabolism	AD	In vivo	/	↑ ACH, GLUT-1 ↓ γ-aminobutyric acid, glutamic acid, IRS-1	[102]
	Protection of cerebral microvessels	AD	In vivo	/	↑ LRP1, GLUT1, GLUT3 ↓ RAGE	[103]
AOLA	Regulation of podocyte autophagy	DN	In vivo	PI3K/Akt/mTOR	↑ Beclin-1, Atg5, LC3-Ⅱ	[105]
	Antiproliferation of mesangial cells	DN	In vitro	PTEN/PI3K/AKT	↓ miRNA-21, p-AKT, AKT, PI3K ↑ PTEN	[106]
AOFV	Analgesia, regulation of the body’s energy metabolism and immune function	Diarrhea	In vivo	/	↑ ATPase, cAMP, IgG, ratio of cAMP/cGMP ↓ TNF-α, NO, 5-HT	[107]
AOAF	Antibacterial, antitumor	/	In vivo	/	↓ staphylococcus aureus, bacillus subtilis, pseudomonas aeruginosa, escherichia coli ↓ transplanted tumors S180 and H22	[108]
	Antioxidant	AD	In vivo	/	↓ Tau, LDH	[109]
AOST	Modulation of immunity, anti-inflammatory	Atopic dermatitis	In vivo	/	↓ IL-4, IL-13, TNF-α, IgE	[110]

(↑ means increase, ↓ means decrease).

## 5. Pharmacokinetics

Zuo et al. orally administered AOF extract to rats at a dose of 0.50 g/kg body weight and randomly divided them into two groups, each placed in a separate metabolic cage. Blood samples were collected before and 10 min, 30 min, and 1, 1.5, 2, 3, 4, 6, 8, 12, 16, 24, and 36 h after administration, and urine and fecal samples were collected at different time points before and 0–24 h after administration. He used UPLC-MS/MS to determine the concentrations of five terpenoids and also used ultra-UHPLC-Q-orbitrap HRMS to identify 27 metabolites of five terpenoids in plasma, urine, and feces of and found that the metabolic pathways were mainly glucoside conjugation, dehydration, desaturation, and glycine conjugation [111].

Zhao established a rapid and simple UHPLC-MS/MS method for determining the content of nootkatone in rat plasma and various tissues at different times before and after administration and found that nootkatone was rapidly distributed through plasma in the heart, liver, spleen, lungs, kidneys, and brain tissue after oral administration and then rapidly eliminated, mainly through the kidneys, and it was able to pass through the blood–brain barrier. Additionally, 34 metabolites were identified in the plasma, urine, and feces of healthy rats administered nootkatone orally by UHPLC-Q- orbitrap HRMS, revealing that nootkatone could be analyzed in healthy rats by UHPLC. The plasma, urine, and feces of rats receiving nootkatone orally were analyzed by UHPLC-Q-orbitrap HRMS, and a total of 34 metabolites were identified, revealing that nootkatone can undergo various complex reactions such as oxidative reaction, dehydration, hydration, desaturation, glucuronide binding, and glycine binding in rats [112].

Wen et al. anesthetized the rats with ether at different time points and killed them with abdominal aortic bleeding and then orally took the AOF extract, finally collecting 14 tissue specimens. He measured the distribution of active ingredients and phase II metabolites of AOF extracts in various tissues of rats after oral administration by using the LC-MS/MS method. The results showed that the active ingredients of AOF were mainly distributed in the gastrointestinal tissue, followed by the liver, with an overall distribution level of stomach > small intestine > large intestine > liver. From a pharmacokinetic perspective, the tissue distribution of active ingredients of AOF partially supports its antidiarrhea effect [113].

Qi et al. compared the pharmacokinetics of the constituents in AOSC herb pairs and the single herbs between healthy and Aβ_1-42_ induced AD model rats. UPLC-MS/MS was used to measure the concentrations of three schisandrin compounds (nootkatone, chrysin, protocatechuic acid) and six schisandrin compounds (schisandrin, deoxyschizandrin, schisandrin B, schisandrin C, gomisin A, and gomisin B). It was found that compared with the oral administration of a single drug, the oral administration of the AOSC herb pair increased the absorption of the above nine compounds and slowed down the elimination of the other eight compounds except gomisin A. In addition, the use of the AOSC herb pair resulted in more absorption and slower elimination of the chemical components in the pair in AD model rats than in healthy rats, which may be attributed to the fact that more herb components enter the blood circulation in AD model rats due to the damage of the intestinal mucosal barrier [114].

In summary, the pharmacokinetic studies on AOF mainly focus on the metabolic pathways and the distribution of metabolites in various tissues. Elucidating the pharmacokinetic characteristics of AOF in vivo can help the further development and utilization of AOF, which provides a basis for the safe and rational use of AOF in the clinic. However, there are still insufficient pharmacokinetic research studies on the other main active ingredients of AOF, and further research studies are needed in the future. HPs, the combination of two herbs, are a form of clinical prescription in Chinese medicine, and the studies of the mechanism of Chinese herb pairs are of great significance in revealing the rationality and scientificity of Chinese medicinal prescriptions. At present, there are few pharmacokinetic studies on the herb pairs performed by comparing the differences in pharmacokinetic parameters between single extracts and mixed extracts of herb pairs, analyzing the interactions between the Chinese medicine components, and elaborating on the possible mechanisms of herb pairs. Furthermore, the pharmacokinetic studies on the use of single compounds in HPs are also insufficient, and the study of the combination of single compounds is good for exploring the potential mechanism of its influence on the compatibility of herb pairs, which is also conducive to the research of TCM.

## 6. Conclusions and Future Perspectives

AOF is widely used in various aspects as a natural medicine with developmental value. Scholars have conducted in-depth research on it, and from 2018 to the present, there has been a wealth of studies on its phytochemistry, isolation methods, pharmacology, pharmacokinetics, and so on. The new compounds isolated from AOF include terpenes, flavonoids, diarylheptanoids, phenolic acid, sterols, alkanes, fats, etc. The isolation methods include the microwave-assisted method, response surface method, HPLC-MRM-MS analytical method, UPLC-Orbitrap-HRMS method, UPLC-MS/MS method, hot water leaching method, ethanol leaching method, and so on. Pharmacological effects include neuroprotection, regulation of metabolic disorders, antioxidant activity, antiapoptosis, anti-inflammatory activity, antidiabetic activity, antihyperuricemia, antiaging, antidiuresis, immune regulation, antitumor activity, renal protection, hepatoprotection, antiasthma, and so on. In addition, in recent years, rich achievements have been made in the study of the herb pairs and pharmacokinetics of AOF. The above research results provide assistance for further research on AOF. However, there is still a lot of work to be conducted in the development and utilization of AOF.

Firstly, the newly isolated compounds in recent years are mainly terpenoids, which may be only a small part of AOF; compounds such as flavonoids, polysaccharides, and other compounds may still have the value of isolation, and it is still recommended to continue the development of new compounds in the future. In addition, the pharmacology and mechanism of action of new compounds extracted and isolated from AOF still need to be further studied, which will be beneficial for the development and utilization of AOF. The present studies are mostly from a class of compounds for separation; how to further isolate a single component from a class of components, optimize the isolation method, and improve the extraction rate are the focus of future research. There is a high demand for *Alpinia oxyphylla*, and other parts of *Alpinia oxyphylla* such as leaves, stems, and shells also contain rich effective ingredients. On the one hand, optimizing the isolation method and improving the obtaining rate are conducive to saving resources and meeting the increasing market demand; on the other hand, it is conducive to deepening the understanding of the whole plant of *Alpinia oxyphylla*, comprehensively evaluating its medicinal value, and improving its economic value.

Secondly, in the past five years, experimental research on the pharmacological effects of AOF has mainly been conducted in vivo, but the proportion of in vitro studies is also increasing. There are relatively few experimental studies directly affecting the human body, indicating that in the future, research can be developed towards human experimental research to provide more clinical references. AOF has a variety of pharmacological effects and has been studied more in recent years in terms of antioxidant, antiapoptotic, and anti-inflammatory properties for the treatment of a wide range of diseases. However, it also has different findings in other aspects. As for the nervous system, in recent years, scholars have conducted more studies on the treatment of neurological disorders with AOF. Relevant studies have shown that AOF can treat AD by regulating metabolic disorders, and its specific mechanism needs to be further studied. In addition, it is still to be confirmed whether the mechanism of AOF in treating other neurological diseases can be studied from the perspective of regulating metabolic disorders. In terms of immunomodulation, AOF is only shown to have a regulatory effect on some immune cells and immune organs, which needs to be further researched in the future. The research on the pharmacological mechanisms of hypoglycemia, antitumor, urinary shrinkage, antiasthma, etc., is not systematic and is incomplete, and its pharmacological effects through the regulation of what signaling pathway is used still need to be further researched.

Thirdly, most of the studies on herb pairs of AOF are on mixed extracts. Clarifying the mechanism of action of the mixed extracts, the ratio between the main active ingredients, and the single compounds is conducive to exploring the potential mechanism of the HPs and deepening the study of AOF. The pharmacokinetic studies of the mixed extracts and the main single components of AOF are still insufficient. In addition, the pharmacokinetic study on the herb pairs is lacking; by comparing the differences in pharmacokinetic parameters between the single extracts and the mixed extracts of herb pairs, it is helpful to analyze the existence of pharmacokinetic interactions, which is conducive to the elaboration of the mechanism of the herb pairs. What is more, pharmacokinetic studies on the use of the single compounds in HPs are also insufficient; the study of the combination of single compounds is good for exploring the potential mechanism of each compound’s influence on the compatibility of herb pairs, which is also conducive to the research of TCM.

In this paper, the medicinal value of AOF is reviewed from the aspects of phyto-chemistry, isolation methods, pharmacology, pharmacokinetics, etc. The research results in these areas are abundant, providing reference for its potential clinical application. However, the research on these aspects is not in-depth and will still benefit from the value of research in the future. AOF deserves further promotion and development as a high-value medicine.

## Figures and Tables

**Table 1:** Terpene compound names and molecular formulas isolated from AOF.

Compound No.	Components	Molecular Formula	Ref.
**1**	1*β*,4*β*,7*β*-trihydroxyeudesmane	C_15_H_28_O_3_	[12]
**2**	bullatantriol	C_15_H_28_O_3_	[12]
**3**	7*α*-hydroperoxy eudesma-3,11-diene-2-one	C_15_H_22_O_3_	[13]
**4**	7*β*-hydroperoxy eudesma-3,11-diene-2-one	C_15_H_22_O_3_	[13]
**5**	3*α*-hydroxynootkatone	C_15_H_22_O_2_	[13]
**6**	oxyphylloneside A	C_26_H_40_O_9_	[14]
**7**	oxyphylloneside B	C_22_H_34_O_11_	[14]
**8**	carvacrol 2-*O*-*β*-glucopyranoside	C_16_H_24_O_6_	[14]
**9**	thymoquinol 2-*O*-*β*-glucopyranoside	C_16_H_24_O_7_	[14]
**10**	(2*R*,5*R*,7*R*,10*S*)-2,7-dihydroxyl-eudesmane-3(4),11(12)-diene	C_15_H_24_O_2_	[15]
**11**	α-rotunol	C_15_H_22_O_2_	[15]
**12**	(1*S*,4*S*,5*R*,7*S*)-1-hydroxyl-eremophilane-9(10),11(12)-diene-8-one	C_15_H_22_O_2_	[15]
**13**	cyperusol A1	C_15_H_22_O_3_	[15]
**14**	(6*R*,9*S*,10*S*)-10-hydroxyl-11,12,13-trinor-cadinane-4(5)-ene-3-one	C_12_H_14_O_2_	[15]
**15**	(4*R*,5*R*,7*R*,10*R*)-12-nor-eudesma-4,5-epoxy-3-one	C_14_H_20_O_3_	[16]
**16**	(3*S*,4*S*,5*R*,7*R*,10*S*)-eudesma-4,5-epoxy-11-en-3,7-diol	C_15_H_24_O_3_	[16]
**17**	(4*S*,5*R*,10*S*)-12-nor-eudesma-6-en-4,5-diol-11-one	C_14_H_22_O_3_	[16]
**18**	(3*S*,4*S*,5*R*,10*R*)-12-nor-eudesma-3,4-epoxy-6-en-5-ol-11-one	C_14_H_20_O_3_	[16]
**19**	(3*R*,4*S*,10*R*)-11,12,13-trinor-eudesma-3,4-epoxy-5-en-7-one	C_12_H_16_O_2_	[16]
**20**	(3*R*,4*S*,10*S*)-11,12,13-trinor-eudesma-3,4-epoxy-5-en-7-one	C_12_H_16_O_2_	[16]
**21**	(6*S*,7*S*,10*R*)-eudesma-6,7-epoxy-4-en-3-one	C_15_H_22_O_2_	[16]
**22**	(5*R*,7*R*,10*S*,11*S*)-eudesma-5,11-epoxy-3-en-12-ol	C_15_H_24_O_2_	[16]
**23**	(5*S*,7*R*,10*S*)-eudesma-3-en-7,11,12-triol	C_15_H_26_O_3_	[16]
**24**	(5*R*,7*R*,10*S*)-eudesma-4(15)-en-7,11,12-triol	C_15_H_26_O_3_	[16]
**25**	(5*R*,7*S*,10*S*)-eudesma-4(15),11-dien-7-ol	C_15_H_24_O	[16]
**26**	(2*R*,5*R*,7*S*,10*S*)-eudesma-4,11-dien-2-ol	C_15_H_24_O	[16]
**27**	(4*R*,5*R*,7*R*,10*R*)-eudesma-11-en-5-ol-1-one	C_15_H_24_O_2_	[16]
**28**	epialpiniol	C_15_H_24_O_2_	[16]
**29**	alpinoxyphllaone A	C_15_H_24_O_3_	[16]
**30**	alpinoxyphllaone B	C_18_H_28_O_4_	[16]
**31**	neoxyphyllanene	C_15_H_22_O_2_	[16]
**32**	oxyphyllin A	C_13_H_20_O_2_	[17]
**33**	oxyphyllin B	C_15_H_22_O_2_	[17]
**34**	oxyphyllin C	C_15_H_22_O_3_	[17]
**35**	oxyphyllin D	C_15_H_22_O_4_	[17]
**36**	oxyphyllin E	C_14_H_18_O_2_	[17]
**37**	oxyphyllin F	C_14_H_18_O_3_	[17]
**38**	oxyphyllin G	C_14_H_20_O_2_	[17]
**39**	oxyphyllin H	C_15_H_22_O_2_	[17]
**40**	oxyphyllin I	C_15_H_24_O_3_	[17]
**41**	oxyphyllin J	C_14_H_22_O_3_	[17]
**42**	(4S*,7S*,9R*)-14-nor-5(10),11(12)-dien-9-ol-1-one-eudesma	C_14_H_20_O_2_	[18]
**43**	cyperusol A4	C_15_H_22_O_3_	[18]
**44**	alpinoxyphyllone C	C_15_H_24_O_2_	[19]
**45**	(3*S*,4*S*,5*R*,7*R*)-eremophila-3,4-epoxy-1(10),11-dien-2-one	C_15_H_20_O_2_	[19]
**46**	(3*R*,4*R*,5*R*,7*R*)-eremophila-3,4-epoxy-1(10),11-dien-2-one	C_15_H_20_O_2_	[19]
**47**	(4*R*,5*S*,7*S*,9*R*)-eremophila-1(10),11-dien-9-ol	C_15_H_24_O	[19]
**48**	(2*R*,4*R*,5*S*,7*S*,9*R*)-eremophila-1(10),11-dien-2,9-diol	C_15_H_24_O_2_	[19]
**49**	(1*R*,2*R*,4*R*,5*S*,7*R*)-eremophila-9,11-dien-1,2-diol	C_15_H_24_O_2_	[19]
**50**	oxyphyllone J	C_14_H_18_O_3_	[19]
**51**	oxyphyllone K	C_14_H_24_O_2_	[19]
**52**	(±)oxyphyllone I	C_15_H_20_O_2_	[19]
**53**	oxyspirone A	C_15_H_24_O_2_	[19]
**54**	oxyspirone B	C_15_H_26_O_2_	[19]
**55**	alpinoxyphyllol A	C_15_H_26_O_2_	[20]
**56**	alpinoxyphyllol B	C_15_H_26_O_2_	[20]
**57**	2-*O*-ethyl-*β*-nootkatol	C_17_H_28_O	[20]
**58**	11-Hydroxyisohanalpinone	C_15_H_22_O_4_	[20]
**59**	6*S*-Oxyphyllenone H	C_14_H_22_O_2_	[20]
**60**	6*R*-Oxyphyllenone H	C_14_H_22_O_2_	[20]
**61**	oxyphylleudne J	C_15_H_23_ClO_3_	[21]
**62**	oxyphyllerene D	C_15_H_22_O_3_	[21]
**63**	oxyphyllerene E	C_15_H_22_O_3_	[21]

**Table 2:** Flavonoid compound names and molecular formulas isolated from AOF.

Compound No.	Components	Molecular Formula	Ref.
**64**	baicalein	C_15_H_10_O_5_	[23]
**65**	wogonin	C_16_H_12_O_5_	[23]
**66**	myricetin	C_15_H_10_O_8_	[23]
**67**	Oxyphyllvonide A	C_24_H_22_O_13_	[24]
**68**	Oxyphyllvonide B	C_24_H_22_O_13_	[24]
**69**	Oxyphyllvonide C	C_24_H_22_O_13_	[24]
**70**	Oxyphyllvonide D	C_24_H_22_O_14_	[24]
**71**	Oxyphyllvonide E	C_24_H_22_O_14_	[24]
**72**	Oxyphyllvonide F	C_24_H_22_O_14_	[24]
**73**	Oxyphyllvonide G	C_23_H_20_O_14_	[24]
**74**	Oxyphyllvonide H	C_26_H_24_O_15_	[24]

**Table 3:** Diarylheptanoid compound names and molecular formulas isolated from AOF.

Compound No.	Components	Molecular Formula	Ref.
**75**	1, 2-benzenedicarboxylic acid	C_26_H_42_O_4_	[12]
**76**	(*E*)-1-(4-hydroxy-3-methoxy-phenyl)-7-(4-hydroxy-phenyl)-hept-4en-3-one	C_20_H_22_O_4_	[12]
**77**	5-hydroxy-7-(4″-hydroxy-3″-methoxyphenyl)-1-phenyl-3-heptanone	C_20_H_24_O_4_	[12]
**78**	dihydrogingerenone B	C_21_H_26_O_6_	[12]
**79**	1,5-epoxy-3-hydroxy-1-(4-hydroxy-3,5-dimethoxyphenyl)-7-(4-hydroxy-3-methoxyphenyl)heptane	C_22_H_28_O_7_	[12]

**Table 4:** Other compound names and molecular formulas isolated from AOF.

Compound No.	Components	Molecular Formula	Ref.
**80**	15-methyl-tetracontane	C_41_H_84_	[12]
**81**	1-tetratriacontanol	C_34_H_70_O	[12]
**82**	bis-(2-ethylhexyl) terephthalate	C_24_H_38_O_4_	[15]
**83**	3-methoxy-4-Hydroxy-diphenylhexane	C_19_H_24_O_2_	[26]
**84**	20-propyl-β-sitosterol	C_32_H_56_O	[26]
**85**	ferulic acid	C_10_H_10_O_4_	[27]

## References

[1] Yan X.X., Ren B.L., Wang M.Y., Wang Q.L., Yang Q., Tang H., Wang Z.N. (2019). Present situation and development strategy of *Alpinia oxyphylla*. China J. Chin. Mater. Med..

[2] Pharmacopoeia of the people’s republic of China (2020). Chinese Pharmacopoeia.

[3] Zhang Q., Zheng Y., Hu X., Hu X., Lv W., Lv D., Chen J., Wu M., Song Q., Shentu J. (2018). Ethnopharmacological uses, phytochemistry, biological activities, and therapeutic applications of *Alpinia oxyphylla* miquel: A review. J. Ethnopharmacol..

[4] Niu Q., Gao Y.M., Liu P.H. (2020). Optimization of microwave-assisted extraction, antioxidant capacity, and characterization of total flavonoids from the leaves of *Alpinia oxyphylla* miq. Prep. Biochem. Biotechnol..

[5] Yang X., Yang Y., Chen H., Xu T., Li C., Zhou R., Gao L., Han M., He X., Chen Y. (2020). Extraction, isolation, immunoregulatory activity, and characterization of *Alpiniae oxyphyllae* fructus polysaccharides. Int. J. Biol. Macromol..

[6] Zhu Y., Ying D.H., Wu Z., Lin D., Xie Y.Q. (2019). Study on optimization of extraction process of total flavonoids from Alpinia oxyphylllae fructus by response surface methodology. Chin. Arch. Tradit. Chin. Med..

[7] Chen Y., Li G., Law H.C.H., Chen H., Lee S.M. (2020). Determination of oxyphylla A enantiomers in the fruits of *Alpinia oxyphylla* by a chiral high-performance liquid chromatography-multiple reaction monitoring-mass spectrometry method and comparison of their in vivo biological activities. J. Agric. Food Chem..

[8] Zhang M.Y., Zuo L.H., Zhou L., Gao Y.Q., Guan K.L., Du X.Y., Zhang R., Jia Q.Q., Pei J.Y., Li H.B. (2020). Analysis and identification of sesquiterpenes in Alpinia oxzphylla miq based on UPLC-Q-Orbitrap HRMS. Chin. Tradit. Herb. Drugs..

[9] Ying L., Wang D., Du G. (2021). Analysis of bioactive components in the fruit, roots, and leaves of *Alpinia oxyphylla* by UPLC-MS/MS. Evid. Based Complement. Altern. Med..

[10] Duan Z.W., Chen T., Chen L., He A., Wang S.P., Xie H. (2021). Optimization of different extraction process and antioxidant activity of polyphenols from *Alpinia oxyphylla* fructus hull. Food Sci. Technol..

[11] Chang H.Y., Cheng T.H., Wang A.H. (2021). Structure, catalysis, and inhibition mechanism of prenyltransferase. IUBMB Life.

[12] Wang Y.L. (2019). Study on Chemical Constituents of the Ethyl Acetate Part of *Alpinia Oxyphylla* Miq. and Their Biological Activity. Master’s Thesis.

[13] Thapa P., Lee Y.J., Nguyen T.T., Piao D., Lee H., Han S., Lee Y.J., Han A.R., Choi H., Jeong J.H. (2021). Eudesmane and eremophilane sesquiterpenes from the fruits of *Alpinia oxyphylla* with protective effects against oxidative stress in adipose-derived mesenchymal stem cells. Molecules..

[14] Zhu Y.T., Chen H., Liu X.N., Li K.Z., Liu S.J., Feng W.S., Cheng Y.X., Wang Y.Z. (2023). Two new terpene glycosides from the *Alpiniae oxyphyllae* fructus. Acta Pharm. Sin..

[15] Qiu C.X., Wang J., Mu L.P., Zhang R.P., Chen X.L. (2023). Chemical constituents from the fruits of *Alpinia oxyphylla* and their neuroprotective effects. Acta Pharm. Sin..

[16] Dong J., Zhou M., Qin Q., Li T., Yao X., Geng J., Yu Y. (2023). Structurally diverse new eudesmane sesquiterpenoids with anti-inflammatory activity from the fruits of *Alpinia oxyphylla*. Bioorg. Chem..

[17] Qiu C., Mu L., Wang J., Tang R., Hou B., Hu W., Zhang R., Chen X. (2023). Sesquiterpenoids from the fruits of *Alpinia oxyphylla* miq. and their neuroprotective effect. Phytochemistry.

[18] Bai W., Wang T., Yang X., Wang Z., Li H., Geng J. (2023). Two new sesquiterpenoids from the fruits of *Alpinia oxyphylla*. Nat. Prod. Res..

[19] Dong J., Zhou M., Pan D.B., Qin Q.Y., Li T., Yao X.S., Li H.B., Yu Y. (2023). Eremophilane and cadinane sesquiterpenoids from the fruits of *Alpinia oxyphylla* and their anti-inflammatory activities. Food Funct..

[20] Cui C., Wu S.L., Chen J.J., Gongpan P., Guan M., Geng C.A. (2023). Sesquiterpenoids from *Alpinia oxyphylla* with GLP-1 stimulative effects through Ca^2+^/CaMKII and PKA pathways and multiple-enzyme inhibition. J. Agric. Food Chem..

[21] Lu B.T., Zhu Y.T., Liu X.N., Niu H.Y., Zhang M.Y., Feng W.S., Wang Y.Z. (2024). Three new sesquiterpenoids from the *Alpinia oxyphyllae* fructus. Acta Pharm. Sin..

[22] Yuan L., Pan K., Li Y., Yi B., Gao B. (2021). Comparative transcriptome analysis of *Alpinia oxyphylla* miq. reveals tissue-specific expression of flavonoid biosynthesis genes. BMC Genom. Data.

[23] Shi Z. (2020). Study on the Chemical Constitutions of the Ethyl Acetate Extract from *Alpinia Oxyphylla* and Activity of the Chrysin. Master’s Thesis.

[24] Zhu Y.T., Fang H.B., Liu X.N., Yan Y.M., Feng W.S., Cheng Y.X., Wang Y.Z. (2023). Unusual acetylated flavonol glucuronides, oxyphyllvonides A-H with renoprotective activities from the fruits of *Alpinae oxyphylla*. Phytochemistry.

[25] Jahng Y., Park J.G. (2018). Recent studies on cyclic 1,7-diarylheptanoids: Their isolation, structures, biological activities, and chemical synthesis. Molecules.

[26] Feng H.M., Wang Z.P., Xu D.P. (2019). Study on various components of *Alpinia oxyphylla* fructus in urine-reducing effect. Food Mach..

[27] Li T.T. (2021). Study on the Chemical Constituents and Related Activities of *Alpinia Oxyphylla* miq. Master’s Thesis.

[28] Xu J., Wang F., Guo J., Xu C., Cao Y., Fang Z., Wang Q. (2020). Pharmacological mechanisms underlying the neuroprotective effects of *Alpinia oxyphylla* Miq. on alzheimer’s disease. Int. J. Mol. Sci..

[29] Duan L.H., Li M., Wang C.B., Wang Q.M., Liu Q.Q., Shang W.F., Shen Y.J., Lin Z.H., Sun T.Y., Wu Z.Z. (2020). Protective effects of organic extracts of *Alpinia oxyphylla* against hydrogen peroxide-induced cytotoxicity in PC12 cells. Neural Regen. Res..

[30] Xu S.Y., Ji X.Y., Shi Z., Chen X., Tan R., Jiang H.Z. (2023). Chemical composition of *Alpinia oxyphylla* miq. and chrysin protective activity on neuron Cells. Pharm. Chem. J..

[31] Zhang M.Y. (2020). Pharmacodynamics and Mechanical Study of *Alpiniae Oxyphyllae* Fructus in the Treatment of Alzheimer’s Disease Based on Metabolomics and Network Pharmacology. Master’s Thesis.

[32] Sun Z., Zhang Y., Zhang M., Zhou S., Cheng W., Xue L., Zhou P., Li X., Zhang Z., Zuo L. (2023). Integrated brain and plasma dual-channel metabolomics to explore the treatment effects of *Alpinia oxyphylla* fructus on alzheimer’s disease. PLoS ONE.

[33] Zhou S., Liu L., Zhang Y., Zhang Z., Li H., Fan F., He J., Kang J., Zuo L. (2023). Integrated untargeted and targeted metabolomics to reveal therapeutic effect and mechanism of *Alpiniae oxyphyllae* fructus on alzheimer’s disease in APP/PS1 mice. Front. Pharmacol..

[34] Ma J.Q. (2019). The Effect and Mechanism of Oil Extract from *Alpinia Oxyphylla* miq. Fruit on Learning and Memory Impairment in. Master’s Thesis.

[35] He B., Xu F., Xiao F., Yan T., Wu B., Bi K., Jia Y. (2018). Neuroprotective effects of nootkatone from *Alpiniae oxyphyllae* fructus against amyloid-β-induced cognitive impairment. Metab. Brain Dis..

[36] Li R., Wang L., Zhang Q., Duan H., Qian D., Yang F., Xia J. (2022). *Alpiniae oxyphyllae* fructus possesses neuroprotective effects on H_2_O_2_ stimulated PC12 cells via regulation of the PI3K/Akt signaling pathway. Front. Pharmacol..

[37] He B., Xu F., Yan T., Xiao F., Wu B., Wang Y., Bi K., Jia Y. (2019). Tectochrysin from *Alpinia oxyphylla* miq. alleviates Aβ1-42 induced learning and memory impairments in mice. Eur. J. Pharmacol..

[38] Bian Y., Chen Y., Wang X., Cui G., Ung C.O.L., Lu J.H., Cong W., Tang B., Lee S.M. (2021). Oxyphylla A ameliorates cognitive deficits and alleviates neuropathology via the Akt-GSK3β and Nrf2-Keap1-HO-1 pathways in vitro and in vivo murine models of alzheimer’s disease. J. Adv. Res..

[39] Vashisth K.M. (2023). The Potential Therapeutic Effect of *Alpinia Oxyphylla* Miquel Formula Granules on Cognitive Impairment in Triple-Transgenic Alzheimer’s Mice. Master’s Thesis.

[40] Wang Y., Wang M., Xu M., Li T., Fan K., Yan T., Xiao F., Bi K., Jia Y. (2018). Nootkatone, a neuroprotective agent from *Alpiniae oxyphyllae* fructus, improves cognitive impairment in lipopolysaccharide-induced mouse model of alzheimer’s disease. Int. Immunopharmacol..

[41] Yan T., Zhang X., Mao Q., Wu B., He B., Jia Y., Shang L. (2022). Alpinae oxyphyllae fructus alleviated LPS-induced cognitive impairments via PI3K/AKT/NF-κB signaling pathway. Environ. Toxicol..

[42] Ji Z.H., Zhao H., Liu C., Yu X.Y. (2020). In-vitro neuroprotective effect and mechanism of 2β-hydroxy-δ-cadinol against amyloid β-induced neuronal apoptosis. Neuroreport.

[43] Wang S., Wang T.T., Zhang H., Shi H.S., Zhao S.J., Piao J.H., Tian J.N. (2021). Exploration on the action mechanism of sharpleaf galangal fruit on alzheimer’s disease based on network pharmacology. Chin. Med. Mod. Distance Edu. Chin..

[44] Yin Y., Zhang Y., Wu X.R., Huo M.K., Jiang L.Z. (2021). Study on the protective effect of serum containing Yizhiren on lipopolysaccharide induced microglial cell injury in mice. Mod. J. Integr. Tradit. Chin. West. Med..

[45] Shi W., Zhong J., Zhang Q., Yan C. (2020). Structural characterization and antineuroinflammatory activity of a novel heteropolysaccharide obtained from the fruits of *Alpinia oxyphylla*. Carbohydr. Polym..

[46] He Y., Chen S., Tsoi B., Qi S., Gu B., Wang Z., Peng C., Shen J. (2021). *Alpinia oxyphylla* miq. and its active compound p-coumaric acid promote brain-derived neurotrophic factor signaling for inducing hippocampal neurogenesis and improving post-cerebral ischemic spatial cognitive functions. Front. Cell Dev. Biol..

[47] Xiao T., Pan M., Wang Y., Huang Y., Tsunoda M., Zhang Y., Wang R., Hu W., Yang H., Li L.S. (2023). In vitro bloodbrain barrier permeability study of four main active ingredients from *Alpiniae oxyphyllae* fructus. J. Pharm. Biomed. Anal..

[48] Xu M., Yang Y., Peng J., Zhang Y., Wu B., He B., Jia Y., Yan T. (2023). Effects of Alpinae oxyphyllae fructus on microglial polarization in a LPS-induced BV2 cells model of neuroinflammation via TREM2. J. Ethnopharmacol..

[49] Zeng P., Liu Y.C., Wang X.M., Ye C.Y., Sun Y.W., Su H.F., Qiu S.W., Li Y.N., Wang Y., Wang Y.C. (2022). Targets and mechanisms of *Alpinia oxyphylla* miquel fruits in treating neurodegenerative dementia. Front. Aging Neurosci..

[50] Morofuji Y., Nakagawa S. (2020). Drug development for central nervous system diseases using in vitro blood-brain barrier models and drug repositioning. Curr. Pharm. Des..

[51] Porsteinsson A.P., Isaacson R.S., Knox S., Sabbagh M.N., Rubino I. (2021). Diagnosis of early alzheimer’s disease: Clinical practice in 2021. J. Prev. Alzheimers Dis..

[52] Li W.J., Xiao S., Zheng Q., Zhu L.Y., Zhang M.X., Yang M., Yan Y., Wang Z.Y. (2021). Mechanism of volatile oil from *Alpinia oxyphylla* in treating alzheimer’s disease based on GC-MS and network pharmacology. China J. Chin. Mater. Med..

[53] GBD 2019 Stroke Collaborators (2021). Global, regional, and national burden of stroke and its risk factors, 1990-2019: A systematic analysis for the global burden of disease study 2019. Lancet Neurol..

[54] Ruscu M., Glavan D., Surugiu R., Doeppner T.R., Hermann D.M., Gresita A., Capitanescu B., Popa-Wagner A. (2024). Pharmacological and stem cell therapy of stroke in animal models: Do they accurately reflect the response of humans?. Exp. Neurol..

[55] Tsai Y.T., Huang H.C., Kao S.T., Chang T.T., Cheng C.Y. (2022). Neuroprotective effects of *Alpinia oxyphylla* miq against mitochondria-related apoptosis by the interactions between upregulated p38 MAPK Signaling and downregulated JNK signaling in the subacute phase of cerebral ischemia-reperfusion in rats. Am. J. Chin. Med..

[56] Cheng C.Y., Chiang S.Y., Kao S.T., Huang S.C. (2021). *Alpinia oxyphylla* miq extract reduces cerebral infarction by downregulating JNK-mediated TLR4/T3JAM- and ASK1-related inflammatory signaling in the acute phase of transient focal cerebral ischemia in rats. Chin. Med..

[57] Wang B., Fang T., Chen H. (2023). Zinc and central nervous system disorders. Nutrients.

[58] Ratcliffe C., Pradeep V., Marson A., Keller S.S., Bonnett L.J. (2024). Clinical prediction models for treatment outcomes in newly diagnosed epilepsy: A systematic review. Epilepsia.

[59] Li J., Jia B., Cheng Y., Song Y., Li Q., Luo C. (2022). Targeting molecular mediators of ferroptosis and oxidative stress for neurological disorders. Oxid. Med. Cell. Longev..

[60] Zhou H., Li S., Li C., Yang X., Li H., Zhong H., Lu J.H., Lee S.M. (2020). Oxyphylla A promotes degradation of α-synuclein for neuroprotection via activation of immunoproteasome. Aging Dis..

[61] Chen Y., Huang Y.T., Liu H.Q., Yang L.Q., Wang Z.Y., Zhang C.C. (2019). Study on the treatment of epileptic rats with ethanol extract of *Alpinia oxyphylla* fructus. World Latest Med. Inf..

[62] Wu B., Gan A., Wang R., Lin F., Yan T., Jia Y. (2023). *Alpinia oxyphylla* miq. volatile oil ameliorates depressive behaviors and inhibits neuroinflammation in CUMS-exposed mice by inhibiting the TLR4-medicated MyD88/NF-κB signaling pathway. J. Chem. Neuroanat..

[63] Xie Y., Xiao M., Ni Y., Jiang S., Feng G., Sang S., Du G. (2018). *Alpinia oxyphylla* miq. extract prevents diabetes in mice by modulating gut microbiota. J. Diabetes Res..

[64] Zong Y.H., Yang K.Y., Yue X.W., Chen Q.J., Sun M., Nie Y.X., Ni Y.L., Xie Y.Q. (2020). The effect of Yizhiren decoction on diabetic nephropathy mice. Chin. Med. Mod. Distance Edu. Chin..

[65] Ni Y.L., Yao Y.J., Wu S., Xie Y.Q. (2023). Study on the protective mechanism of Yizhiren regulating lipid metabolism in mice with diabetic nephropathy. J. Hainan Med. Univ..

[66] Ni Y.L. (2019). Studies on the Mechanism of Yizhiren in the Treatment of Diabetic Nephropathy Based on Gut Microbiota and Metabonomics Analysis. Master’s Thesis.

[67] Jia A., Huang X.Q., Liu S.H., Chang Z.G., Wang D.L., Tan Y.F., Li Y.H. (2023). Regulation of intestinal micro ecology between raw and salt-processed *Alpinia oxyphylla* on renal injury rats. Pak. J. Pharm. Sci..

[68] Sung Y.Y., Kim D.S. (2022). Synergistic impacts of *Alpinia oxyphylla* seed extract and allopurinol against experimental hyperuricemia. BioMed Res. Int..

[69] Lee Y.S., Sung Y.Y., Yuk H.J., Son E., Lee S., Kim J.S., Kim D.S. (2019). Anti-hyperuricemic effect of *Alpinia oxyphylla* seed extract by enhancing uric acid excretion in the kidney. Phytomedicine.

[70] Yang F., Xiao M., Chen B.C., Niu K., Xie Y.Q. (2022). Mechanism study of Yizhiren (*Alpinia oxyphylla*) extract on senescence of caenorhabditis elegans. Chin. Arch. Tradit. Chin. Med..

[71] Xiao M., Chen B., Niu K., Long Z., Yang F., Xie Y. (2022). Alpiniae oxyphylla fructus extract promotes longevity and stress resistance of C. elegans via DAF-16 and SKN-1. Front. Pharmacol..

[72] Chang Y.M., Shibu M.A., Chen C.S., Tamilselvi S., Tsai C.T., Tsai C.C., Kumar K.A., Lin H.J., Mahalakshmi B., Kuo W.W. (2021). Adipose derived mesenchymal stem cells along with *Alpinia oxyphylla* extract alleviate mitochondria-mediated cardiac apoptosis in aging models and cardiac function in aging rats. J. Ethnopharmacol..

[73] Lin H.J., Ramesh S., Chang Y.M., Tsai C.T., Tsai C.C., Shibu M.A., Tamilselvi S., Mahalakshmi B., Kuo W.W., Huang C.Y. (2021). D-galactose-induced toxicity associated senescence mitigated by *Alpinate oxyphyllae* fructus fortified adipose-derived mesenchymal stem cells. Environ. Toxicol..

[74] Lu M., Tan L., Zhou X.G., Yang Z.L., Zhu Q., Chen J.N., Luo H.R., Wu G.S. (2020). Tectochrysin increases stress resistance and extends the lifespan of caenorhabditis elegans via FOXO/DAF-16. Biogerontology.

[75] Chang Y.M., Tamilselvi S., Lin H.J., Tsai C.C., Lin Y.M., Day C.H., Viswanadha V.P., Chang H.N., Kuo W.W., Huang C.Y. (2019). *Alpinia oxyphylla* miq extract ameliorates cardiac fibrosis associated with D-galactose induced aging in rats. Environ. Toxicol..

[76] Kim H.H., Park Y.S., Kim B.K., Ahn H.S. (2019). Beneficial Effects of *Alpiniae oxyphyllae* fructus (AOF)on male reproductive function. Indian J Public Health Res. Dev..

[77] Tie Y., Sun Z., Tong X., Cheng M., Wu Y., Shi Z., Xu P., Xue M., Xu L., Zhou X. (2024). Multi-omic analysis revealed the therapeutic mechanisms of *Alpinia oxyphylla* fructus water extract against bladder overactivity in spontaneously hypertensive rats. Phytomedicine.

[78] Su M.S., Xue S.C., Xu L., Ren X.K., Huang N., Tang Z.Q., Xu M.H. (2020). Effect of intermittent hypoxia on bladder detrusor cell apoptosis and regulatory mechanism of *Alpiniae oxyphyllae* fructus. Chin. J. Pathophysiol..

[79] Han Y., Wu J., Liu Y., Qi J., Wang C., Yu T., Xia Y., Li H. (2019). Therapeutic effect and mechanism of polysaccharide from *Alpiniae oxyphyllae* fructus on urinary incontinence. Int. J. Biol. Macromol..

[80] Su M.S., Xu L., Gu S.G., Huang N., Ren X.K., Cai X.H., Li C.C. (2020). Therapeutic effects and modulatory mechanism of *Alpiniae oxyphyllae* fructus in chronic intermittent hypoxia induced enuresis in rats. Sleep Breath..

[81] Liu T.H. (2019). Immunomodulatory effects of *Alpiniae oxyphyllae* fructus in rats. Clin. Lab J. (Electron. Ed.)..

[82] Tian J.X., Zhong J.W. (2018). Studies on the immunomodulatory effects of *Alpiniae oxyphyllae* fructus on mice. World Latest Med. Inf..

[83] Yang X., Zhou S., Li H., An J., Li C., Zhou R., Teng L., Zhu Y., Liao S., Yang Y. (2021). Structural characterization of *Alpiniae oxyphyllae* fructus polysaccharide 2 and its activation effects on RAW264.7 macrophages. Int. Immunopharmacol..

[84] Busayo F.K., Yang J.L., Ding X.P., Wang Y.L., Gai C.J., Wu F., Dai H.F., Mei W.L., Chen H.Q. (2024). Identification of volatile compounds and their bioactivities from unpolar fraction of *Alpinia oxyphylla* miq. and mining key genes of nootkatone biosynthesis. Nat. Prod. Res..

[85] Yu S.H., Kim H.J., Jeon S.Y., Kim M.R., Lee B.S., Lee J.J., Kim D.S., Lee Y.C. (2020). Anti-inflammatory and anti-nociceptive activities of *Alpinia oxyphylla* miquel extracts in animal models. J. Ethnopharmacol..

[86] Jeon S.Y., Yu S.H., Lee B.S., Kim H.J., Kim C.G., Jang E.J., Lee J.J., Kim D.S., Kim M.R. (2021). Chondroprotective effect of *Alpinia oxyphylla* extract in experimentally induced cartilage degradation in rabbit articular cartilage explants. J. Food Biochem..

[87] Lee Y.M., Son E., Kim S.H., Kim D.S. (2019). Effect of *Alpinia oxyphylla* extract in vitro and in a monosodium iodoacetate-induced osteoarthritis rat model. Phytomedicine.

[88] Chen Y.Y., Chen Y., He Z.T., Wu H.Z. (2020). Experimental study of ethyl acetate extract of *Alpinia oxyphylla* on TFK-1 cell line of cholangiocarcinoma. Chin. J. Clin. Pharmacol..

[89] Yoo E., Lee J., Lertpatipanpong P., Ryu J., Kim C.T., Park E.Y., Baek S.J. (2020). Anti-proliferative activity of A. Oxyphylla and its bioactive constituent nootkatone in colorectal cancer cells. BMC Cancer.

[90] Hui F., Qin X., Zhang Q., Li R., Liu M., Ren T., Zhao M., Zhao Q. (2019). *Alpinia oxyphylla* oil induces apoptosis of hepatocellular carcinoma cells via PI3K/Akt pathway in vitro and in vivo. Biomed. Pharmacother..

[91] Dai C., Liu M., Zhang Q., Das Gupta S., Tang S., Shen J. (2023). Nootkatone supplementation attenuates carbon tetrachloride exposure-induced nephrotoxicity in mice. Antioxidants.

[92] Zhang Y., Huang Z.B., Zhou Z., Ma N., Wang R.Q., Chen M.M., He X.W., Dong L., Xia Z.X., Liu Q. (2022). Nootkatone, a sesquiterpene ketone from *Alpiniae oxyphyllae* fructus, ameliorates metabolic-associated fatty liver by regulating AMPK and MAPK signaling. Front. Pharmacol..

[93] Park C.L., Kim J.H., Jeon J.S., Lee J.H., Zhang K., Guo S., Lee D.H., Gao E.M., Son R.H., Kim Y.M. (2022). Protective effect of *Alpinia oxyphylla* fruit against tert-butyl hydroperoxide-induced toxicity in HepG2 cells via Nrf2 activation and free radical scavenging and its active molecules. Antioxidants.

[94] Fang L., Yan Y., Xu Z., He Z., Zhou S., Jiang X., Wu F., Yuan X., Zhang T., Yu D. (2021). Tectochrysin ameliorates murine allergic airway inflammation by suppressing Th2 response and oxidative stress. Eur. J. Pharmacol..

[95] Wang S., Hu Y., Tan W., Wu X., Chen R., Cao J., Chen M., Wang Y. (2012). Compatibility art of traditional Chinese medicine: From the perspective of herb pairs. J. Ethnopharmacol..

[96] Wang M., Lin F., Zhang X., Zhang M., Yan T., Wu B., Du Y., He B., Jia Y. (2022). Combination of *Alpinia oxyphylla* fructus and Schisandra chinensis fructus ameliorates aluminum-induced alzheimer’s disease via reducing BACE1 expression. J. Chem. Neuroanat..

[97] Qi Y., Cheng X., Jing H., Yan T., Xiao F., Wu B., Bi K., Jia Y. (2019). Effect of *Alpinia oxyphylla*-Schisandra chinensis herb pair on inflammation and apoptosis in alzheimer’s disease mice model. J. Ethnopharmacol..

[98] Qi Y., Jing H., Cheng X., Yan T., Xiao F., Wu B., Bi K., Jia Y. (2020). *Alpinia oxyphylla*-Schisandra chinensis herb pair alleviates amyloid-β induced cognitive deficits via PI3K/Akt/Gsk-3β/CREB pathway. Neuromolecular Med..

[99] Qi Y., Cheng X., Jing H., Yan T., Xiao F., Wu B., Bi K., Jia Y. (2019). Combination of schisandrin and nootkatone exerts neuroprotective effect in alzheimer’s disease mice model. Metab. Brain Dis..

[100] Qi Y., Cheng X., Gong G., Yan T., Du Y., Wu B., Bi K., Jia Y. (2020). Synergistic neuroprotective effect of schisandrin and nootkatone on regulating inflammation, apoptosis and autophagy via the PI3K/AKT pathway. Food Funct..

[101] Wang M., Bi W., Fan K., Li T., Yan T., Xiao F., He B., Bi K., Jia Y. (2018). Ameliorating effect of *Alpinia oxyphylla*-Schisandra chinensis herb pair on cognitive impairment in a mouse model of alzheimer’s disease. Biomed. Pharmacother..

[102] Luo J. (2019). Comparison of Extraction Process of Acorus Tatarinowii-*Alpinia Oxyphylla* miq Essential Oil and Study on the Effect of Inhalation Administration on AD. Master’s Thesis.

[103] Li W.J. (2021). Effect of Volatile Oil from Acorus Tatarinowii and *Alpinia Oxyphylla* on hCMEC/D3 Cells Injured by Aβ25-35 and Preparation of Microemulsion. Master’s Thesis.

[104] Yin D.H., Song L., Xie Y.Q., Zhu Y., Wu Z. (2021). Protective effects of a combination of *Alpinia oxyphylla* fructus and Lindera aggregata on podocytes of mice with diabetic nephropathy through modulation of cellular autophagy. Lishizhen Med. Mater. Med. Res..

[105] Yin D.H., Tang S.Y., Wu Z., Chen Y.Q., Zhu Y. (2024). Study on mechanism of Yizhiren(*Alpiniae oxyphyllae* fructus)-Wuyao(Linderae Radix) drug pair in regulating PI3K/Akt/mTOR pathway-mediated cellular autophagy to protect podocytes. Chin. Arch. Tradit. Chin. Med..

[106] Mao L.Q., Huang L., Xiao M., Du G.K., Yao Y.J., Li X., Tang C., Xie Y.Q., Ni Y.L. (2018). Inhibitory effect of Yizhiren combined with Obtusiloba on proliferation of high glucose-cultured mesangial cells. Shandong Med. J..

[107] Xu R.Y., Dou S.R., Cao Y.G., Tian L.Q., Zhu J.G., Li H.W., Li K., Feng W.S. (2023). The effect of salt-processing on the antidiarrheal efficacy of *Alpiniae oxyphyllae* fructus-Foeniculi fructus medicines. Chin. J. Hosp. Pharm..

[108] Gao L.L., Wang Q., Zhang J.W., Huang R.Q., Zhang X.W. (2019). Study on purification and antibacterial and antitumor activity of flavonoids from Amomum villosum lour and *Alpinia oxyphylla* miq. J. Food Saf. Qual..

[109] Lin W.X., Huang L.P., Deng M.Z., Wang N.B., Lin M.Q., Ma R.X. (2018). Effect of Component of Fructus *Alpiniae oxyphyllae* and Amomum Villosum on alzheimer’s disease cell model induced by okadaic acid. Chin. J. Chin. Med..

[110] Zhang T., Qiu J., Wu X., Huang S., Yuan H., Park S. (2020). Schizonepeta tenuifolia with *Alpinia oxyphylla* alleviates atopic dermatitis and improves the gut microbiome in Nc/Nga mice. Pharmaceutics.

[111] Zuo L., Li J., Xue L., Jia Q., Li Z., Zhang M., Zhao M., Wang M., Kang J., Du S. (2021). Integrated UPLC-MS/MS and UHPLC-Q-orbitrap HRMS analysis to reveal pharmacokinetics and metabolism of five terpenoids from *Alpiniae oxyphyllae* fructus in rats. Curr. Drug Metab..

[112] Zhao M.F. (2022). Pharmacokinetics of Nootkatone *Alpinia Oxyphylla* Fructus in Rats. Master’s Thesis.

[113] Wen Q., Li H.L., Mai S.Y., Tan Y.F., Chen F. (2019). Tissue distribution of active principles from *Alpiniae oxyphyllae* fructus extract: An experimental study in rats. Curr. Pharm. Anal..

[114] Qi Y., Cheng X., Jing H., Yan T., Xiao F., Wu B., Bi K., Jia Y. (2020). Comparative pharmacokinetic study of the components in *Alpinia oxyphylla* miq.-Schisandra chinensis (Turcz.) Baill. herb pair and its single herb between normal and alzheimer’s disease rats by UPLC-MS/MS. J. Pharm. Biomed. Anal..

